# Loss of PFKFB4 induces cell death in mitotically arrested ovarian cancer cells

**DOI:** 10.18632/oncotarget.14910

**Published:** 2017-01-31

**Authors:** Charlotte Taylor, David Mannion, Fabrizio Mirand a, Mohammad Karaminejadranjbar, Sandra Herrero-Gonzalez, Karin Hellner, Yiyan Zheng, Geoffrey Bartholomeusz, Robert C. Bast, Ahmed Ashour Ahmed

**Affiliations:** ^1^ Ovarian Cancer Cell Laboratory, Weatherall Institute of Molecular Medicine, University of Oxford, Headington, Oxford, OX3 9DS, UK; ^2^ Nuffield Department of Obstetrics and Gynaecology, University of Oxford, Women's Centre, John Radcliffe Hospital, Oxford, OX3 9DU, UK; ^3^ Department of Experimental Therapeutics, M.D. Anderson Cancer Center, University of Texas, Houston, TX 77030, USA

**Keywords:** ovarian cancer, paclitaxel, mitotic arrest, PFKFB4, PFKFB3

## Abstract

Taxanes represent some of the most commonly used chemotherapeutic agents for ovarian cancer treatment. However, they are only effective in approximately 40% of patients. Novel therapeutic strategies are required to potentiate their effect and improve patient outcome. A hallmark of many cancers is the constitutive activation of the PI3K/AKT pathway, which drives cell survival and metabolism. We discovered a striking decrease in AKT activity coupled with a significant reduction in glucose 6-phosphate and ATP levels during mitotic arrest in the majority of ovarian cancer cell lines tested, indicating a potential metabolic vulnerability. A high-content siRNA screen to detect novel metabolic targets in mitotically arrested ovarian cancer cells identified the glycolytic enzyme PFKFB4. PFKFB4 depletion increased caspase 3/7 activity, and levels of reactive oxygen species only in mitotically arrested cells, and significantly enhanced mitotic cell death after paclitaxel treatment. Depletion of PFKFB3 demonstrated a similar phenotype. The observation that some ovarian cancer cells lose AKT activity during mitotic arrest and become vulnerable to metabolic targeting is a new concept in cancer therapy. Thus, combining mitotic-targeted therapies with glycolytic inhibitors may act to potentiate the effects of antimitotics in ovarian cancer through mitosis-specific cell death.

## INTRODUCTION

Ovarian cancer is the fifth most common cause of cancer death in women, with the five-year survival rate currently around 41% [1]. Antimitotics, such as taxanes, represent some of the most common chemotherapeutic agents used in the treatment of ovarian cancer. In spite of prolonged mitotic arrest following exposure, a significant proportion of cancer cells escape mitotic catastrophe and develop chemotherapy resistance [2]. Novel therapeutic strategies are required to potentiate the effects of current antimitotic therapies in ovarian cancer to improve patient prognosis [3, 4].

It is well recognised that many cancer cells exhibit an altered metabolism compared to non-malignant cells. While non-malignant cells use the TCA cycle and oxidative phosphorylation as the main source of ATP when oxygen is readily available, malignant cells use glycolysis as their primary energy source, even in oxygen rich conditions, which is known as aerobic glycolysis [5, 6]. This persistent utilisation of aerobic glycolysis is considered a hallmark of many cancers and is known as the Warburg effect [7]. The dependence of cancer cells on aerobic glycolysis to support their high energetic and biosynthetic demands provides a potential therapeutic opportunity, which is exploited by a number of compounds such as lonidamine, Imatinib and 2-deoxyglucose, all of which suppress aerobic glycolysis through their inhibitory effect on the glycolytic enzyme hexokinase [8]. The increased utilisation of glycolysis in cancer cells also imposes a high demand for cellular glucose. This requirement is met by an increase in glucose uptake and breakdown, which is largely stimulated by the enhanced activity of oncogenes. One oncogene, known as AKT, is constitutively active in a large number of cancers, including ovarian cancer, and acts to promote cell survival, growth, and glycolysis [9]. AKT can enhance glucose uptake via the phosphorylation of the Rab GTPase-activating proteins TBC1 domain family 1 (TBC1D1) and AKT substrate of 160kDa (AS160/TBC1D4) [10, 11], while it also directly stimulates the glycolytic enzyme 6-phosphofructo-2-kinase [12] and promotes association of hexokinase with the mitochondria [13]. Given the influence that AKT activity has on glycolysis, investigating AKT signalling and glycolytic activity in ovarian cancer cells during mitotic arrest could lead to the identification of novel metabolic targets that are critical for ovarian cancer cell survival following treatment with mitotic-targeted therapies.

Here we identify a metabolic vulnerability in the majority of ovarian cancer cell lines during mitotic arrest, which arises from a striking decrease in AKT activity, a reduction in glucose 6-phosphate, and a significant decrease in ATP levels. In addition, we have shown that depletion of the glycolytic enzymes PFKFB3 and PFKFB4 prior to paclitaxel treatment can increase cell death in mitotically arrested cells possessing this identified vulnerability. The observation that many ovarian cancer cells lose AKT activity in mitosis and become vulnerable to metabolic targeting is a new concept in cancer therapy. Moreover, we have identified a previously unrecognised role of PFKFB3 and PFKFB4 in mitotically arrested ovarian cancer cell survival. Accordingly, the combination of antimitotics and glycolytic inhibitors may provide a means to enhance patient response to antimitotic therapies in ovarian cancer.

## RESULTS

### The majority of ovarian cancer cell lines exhibit reduced AKT activity and lower ATP levels during mitosis

**Figure 1:**
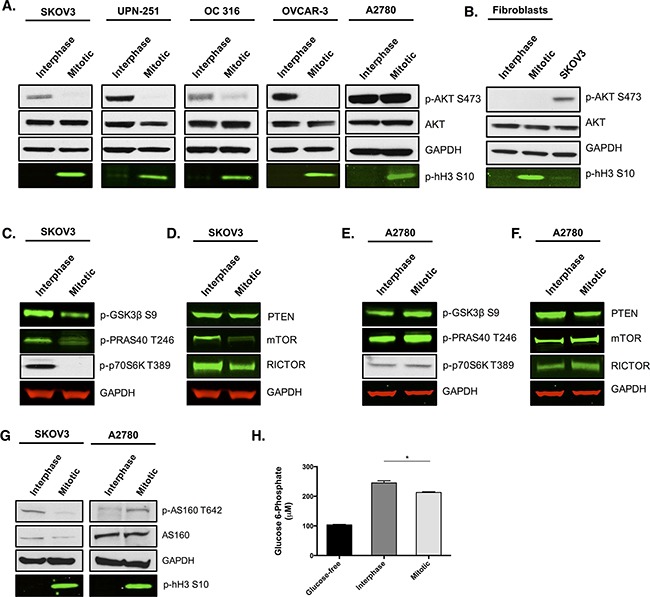
AKT activity is reduced in multiple ovarian cancer cell lines and skin fibroblasts during paclitaxel-induced mitotic arrest Cells were treated with 50 nM paclitaxel for 16 h and separated by mitotic shake-off into the mitotic and interphase populations. **A**. Western blotting was used to determine total AKT expression and AKT phosphorylation at S473 in ovarian cancer cells and **B**. human skin fibroblasts. **C, D**. The phosphorylation of substrates downstream of AKT and the protein expression of key upstream components were also assessed by western-blotting in SKOV3 cells and **E, F**. A2780 cells. **G**. Analysis of AS160 protein expression and its phosphorylation at T642 was examined by western blotting. In the western blots, GAPDH is the loading control, phospho-histone H3 S10 is the mitotic marker and images represent one of three experiments. **H**. Glucose 6-phosphate levels were analysed in SKOV3 cells and presented as the mean +SEM of three experiments.

Whilst investigating signalling alterations in ovarian cancer cells during mitotic arrest, we observed a striking loss of AKT phosphorylation at S473 in the majority of ovarian cancer cell lines tested (Figure 1A). Loss of AKT phosphorylation did not appear to be paclitaxel-dependent as it was present in both spontaneous mitosis and paclitaxel-induced mitotic arrest (Supplementary Figure 1A). Analysis of *AKT* mRNA levels in mitotic cells displayed no change in *AKT1* expression, a slight decrease in *AKT2* and a 50% increase in *AKT3* compared to interphase cells (Supplementary Figure 1B). However, the pan phospho-specific antibodies used in the western blot analysis strongly suggested that the apparent increase in *AKT3* mRNA level is not accompanied by a significant increase in AKT phosphorylation or activity. To determine the phosphorylation status of AKT in non-tumourgenic cells during mitotic arrest, mitotic cells were isolated from human skin fibroblasts following paclitaxel treatment (Figure 1B). AKT phosphorylation at S473 was not observed in either the interphase or mitotic fibroblast cells, suggesting that these cells possess very low levels of AKT phosphorylation compared to SKOV3 cells.

Loss of AKT phosphorylation at S473 in mitotically arrested ovarian cancer cells was accompanied by a significant decrease in AKT activity, since the phosphorylation of several downstream targets of AKT, such as GSK3β, PRAS40 and p70S6K, was reduced (Figure 1C). We also observed a significant reduction in the protein levels of mTOR and RICTOR in mitotically arrested cells (Figure 1D), both of which are known to be essential components of the mTORC2 complex that is important for phosphorylation and activation of AKT [14, 15]. This may explain, at least in part, the loss of AKT phosphorylation during mitosis as it has previously been shown that a reduction in either RICTOR or mTORC2 proteins can prevent phosphorylation of AKT at S473 [15]. We also found that the protein levels of the unphosphorylated and active form of the tumour suppressor PTEN remained unchanged between interphase cells and mitotically arrested cells. Given that PTEN negatively regulates AKT signaling, we concluded that PTEN is unlikely to contribute to the observed decrease in AKT activity in mitotically arrested ovarian cancer cells.

One ovarian cancer cell line, A2780, displayed no change in AKT phosphorylation between interphase and mitotic arrest. Moreover, signalling through AKT in that cell line appeared not to be affected, with the phosphorylation of GSK3β, PRAS40 and p70S6K being maintained, or slightly increased, in the mitotically arrested population (Figure 1E). Contrary to other ovarian cancer cell lines, protein expression of the mTORC2 complex components, mTOR and RICTOR, displayed a modest increase rather than a decrease during mitotic arrest when compared to interphase cells (Figure 1F). Additionally, protein levels of PTEN were decreased during mitotic arrest compared to interphase cells. Therefore, it is possible that the increase in mTORC2 activity coupled with a decrease in active PTEN could explain the maintenance, or modest increase, in AKT signalling during mitotic arrest in A2780 cells. Moreover, a 9 nucleotide deletion spanning the PTEN phosphatase domain has been identified in A2780 cells [16]. This does not affect *PTEN* mRNA expression, but is thought to generate a hypomorphic PTEN protein [17], which could also contribute to the maintenance of AKT signalling in A2780 cells.

The loss of AKT activity in the majority of ovarian cancer cell lines during mitosis was provocative because of the key role that AKT has in driving cancer cell metabolism, survival, and proliferation [18, 19]. The ovarian cancer cell lines SKOV3, OC 316, A2780, UPN-251, and OVCAR-3 have all been shown to possess *PIK3CA* mutations or copy number gain [20–22], which cause the constitutive activation of AKT. The maintenance of PI3K/AKT activation could lead to the phenomenon known as ‘oncogene addiction’ [23], whereby the cancer cell becomes dependent on the enhanced activity of the PI3K/AKT pathway [24]. Therefore, we hypothesised that the observed temporary withdrawal of AKT activity during mitosis might render cancer cells metabolically vulnerable, and that treatment with paclitaxel could extend this therapeutic window. Consistent with this hypothesis was the observation that the phosphorylation of AS160, a direct substrate of AKT that links insulin signaling with the glucose transporter GLUT4 [25], was decreased in mitotically arrested SKOV3 cells, but not in A2780 cells, which instead displayed an increase in AS160 phosphorylation during mitotic arrest (Figure 1G). This implied a reduction in the translocation of GLUT4 to the membrane during mitotic arrest and a consequent reduction in glucose transport. As expected, glucose 6-phosphate levels were significantly decreased in mitotically arrested SKOV3 cells compared to interphase cells (Figure 1H, *p=*0.0268, *t*-test).

**Figure 2:**
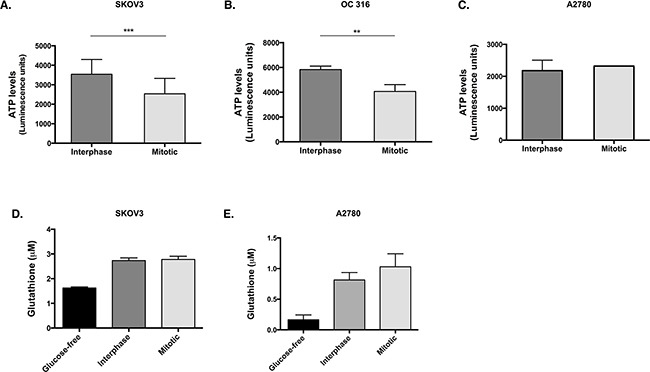
ATP levels are reduced and GSH levels are unchanged in cells that lose AKT activity during paclitaxel-induced mitotic arrest Cells were treated with 50 nM paclitaxel for 16 h and separated by mitotic shake-off into the mitotic and interphase populations. **A**. After counting the cells in the presence of trypan blue and dilution to the correct concentrations, ATP levels were measured using the CellTiter-Glo assay in SKOV3 cells, **B**. OC 316 cells and **C**. A2780 cells. **D**. Glutathione (GSH) levels were assessed using the GSH-Glo Glutathione assay in SKOV3 cells and **E**. A2780 cells. Data are expressed as the mean +SEM of three experiments.

We next hypothesised that the reduction in glucose 6-phosphate would hinder its utilisation in glycolysis and the pentose phosphate pathway. Therefore, the levels of ATP and glutathione (GSH) were investigated as a readout of the flux through glycolysis and the pentose phosphate pathway, respectively. A significant decrease in ATP was observed during mitotic arrest in SKOV3 cells (Figure 2A, *p=*0.005, *t*-test) and OC 316 cells (Figure 2B, *p=*0.0079, *t*-test) compared to interphase cells, while the levels of ATP (Figure 2C) in A2780 cells remained unchanged between the mitotically arrested and interphase populations. Moreover, no significant difference was found in the GSH levels between the mitotically arrested and interphase populations in either SKOV3 or A2780 cells (Figure 2D, 2E).

### A high content kinome screen identifies proteins that are critical for survival in mitotically arrested ovarian cancer cells

**Figure 3:**
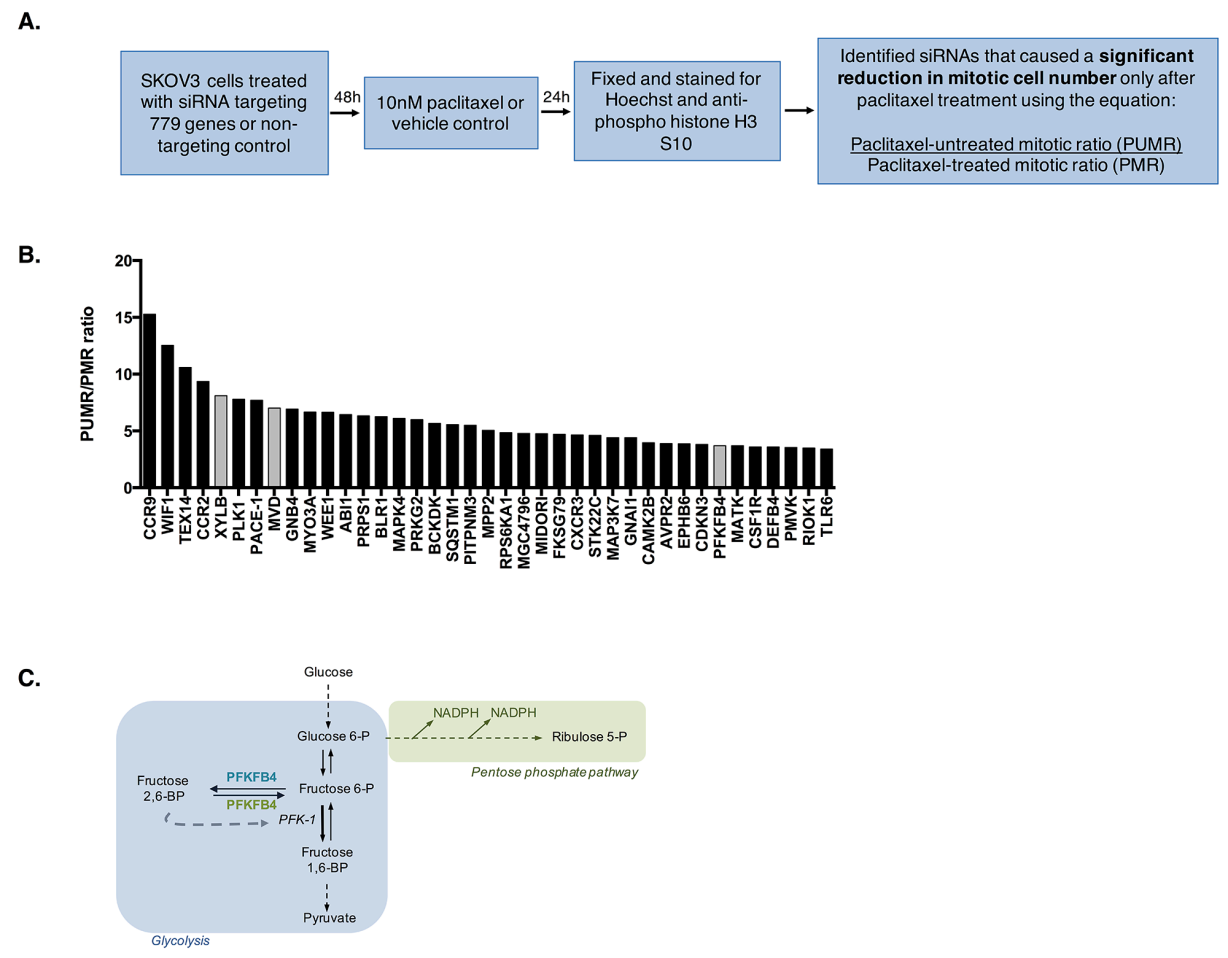
High-content siRNA kinome screen identifies PFKFB4 as important for ovarian cancer cell survival during paclitaxel-induced mitotic arrest **A**. Schematic representation of the screen protocol. The paclitaxel treated mitotic ratio (PMR) was calculated as the mitotic index following gene depletion in paclitaxel-treated cells divided by the mitotic index following non-targeting siRNA control treatment in paclitaxel treated cells. The paclitaxel-untreated mitotic ratio (PUMR) is the mitotic index following gene depletion in untreated cells divided by the mitotic index following non-targeting siRNA control treatment in untreated cells. **B**. A bar plot showing the siRNA targets resulting in a PUMR/PMR ratio of 3 or greater, with metabolic targets highlighted in grey. **C**. Schematic demonstrating the function of PFKFB4 in glycolysis.

The data so far indicate a vulnerability in SKOV3 cells, and potentially other ovarian cancer cell lines, during paclitaxel-induced mitotic arrest. This vulnerability is predicted to arise from a reduction in AKT activity, glucose 6-phosphate, and ATP. We hypothesized that interfering with key metabolic enzymes in these cells might exploit this vulnerability and enhance cell death during paclitaxel-induced mitotic arrest. To investigate this, we conducted a high-content siRNA kinome screen to identify potential metabolic targets that are important for cell survival following mitotic arrest by paclitaxel treatment (Figure 3A). The screen was performed using SKOV3 cells, as they possessed the potential vulnerability during mitotic arrest, and with 10 nM paclitaxel, as this was the optimal concentration to achieve the maximum number of mitotic cells after 24 h without inducing significant cell death, thus providing a window for mitotic cell depletion. Cells were fixed and stained for Hoechst to measure DNA content and anti-phospho-histone H3 S10 to estimate the number of mitotic cells. Depletion of a gene could result in low mitotic index (percentage of mitotic cells compared to all cells) by inducing cell cycle arrest prior to mitosis, interfering with the mitotic checkpoint, or inducing mitotic cell death. We reasoned that a true positive hit would be one that induces cell death in mitosis with minimum interference to mitotic checkpoints or G2 to M transition. Therefore, depletion of such a protein would specifically reduce the paclitaxel treated mitotic ratio (PMR), measured as the mitotic index following gene depletion in paclitaxel treated cells divided by the mitotic index following non-targeting siRNA control treatment in paclitaxel treated cells, as these cells are enriched in mitosis, but have limited effect on the paclitaxel untreated mitotic ratio (PUMR). Therefore, we computed the ratio of PUMR/PMR and considered a cut-off of 3-fold as a positive hit for further validation (Supplementary Table 1). The screen was conducted in triplicate for each condition (9 in total) and siRNA targeting *PLK1* was used as a transfection control as PLK1 depletion is known to be fatal to cells [26, 27].

Using the above mentioned criteria, a number of proteins were identified as causing a significant fold decrease in the number of mitotically arrested cells following paclitaxel treatment after their depletion (Figure 3B). These included two chemokine receptors (CCR), CCR9 and CCR2, which have been implicated in driving oncogenesis and shown to induce apoptosis when depleted in cancerous cells [28, 29], Testis expressed 14 (TEX14) that is a PLK1 regulated protein [30], as well as Wnt inhibitory factor 1 (WIF1), which antagonises Wnt signaling [31] and may therefore be important for the control of the pathway in ovarian cancer cells. In addition, Three enzymes with roles in metabolism were identified from the screen as causing a significant fold decrease in the number of mitotically arrested cells following paclitaxel treatment after their depletion, two of which were more directly linked to glycolysis; XYLB and PFKFB4. Mevalonate diphosphate decarboxylase (MVD) was also found to cause a significant decrease in the number of cells undergoing spontaneous mitosis, indicating that the effects of its depletion were not specific to cells undergoing prolonged mitotic arrest. For this reason, MVD was not selected for further study. Preliminary experiments investigating XYLB depletion could not validate the results of the screen, resulting in phosphofructo-2-kinase/fructose 2,6-bisphosphatase (PFKFB4) being followed up as the only high priority candidate.

**Figure 4:**
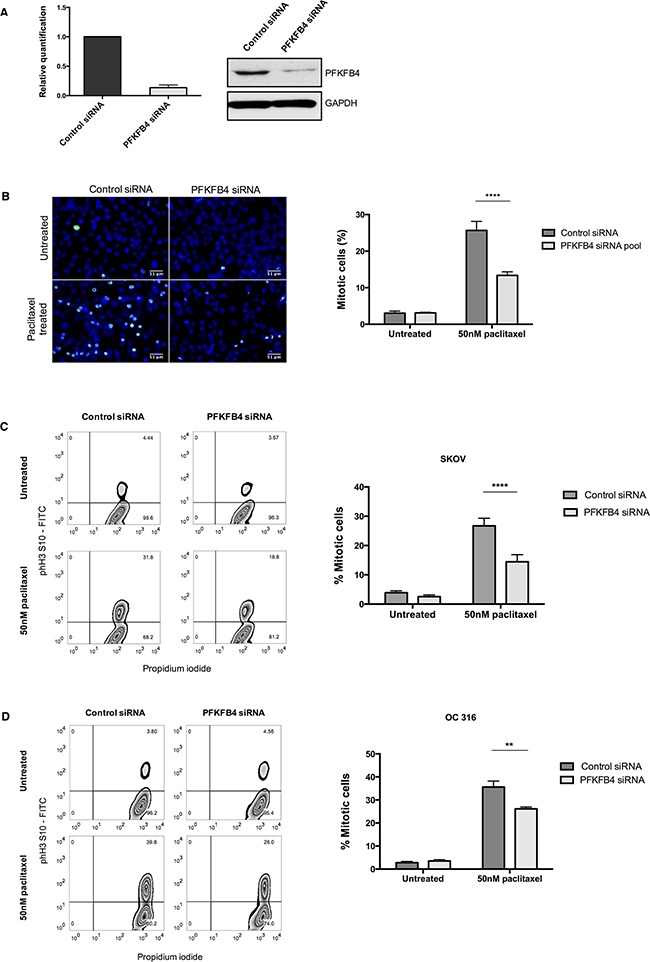
Validation of the screen hit PFKFB4 **A**. *PFKFB4* mRNA and protein levels were assessed by qPCR and western blotting after PFKFB4 depletion using pooled siRNA duplexes in SKOV3 cells for 72 h. **B**. After siRNA-mediated depletion of *PFKFB4* for 72 h followed by 50 nM paclitaxel for 16 h, SKOV3 cells were fixed and stained for immunofluorescence. Images are shown with Hoechst stain (blue) and phospho-histone H3 (S10) (green) and the percentage of mitotically arrested cells were quantified as the mean +SEM of three experiments. **C**. The percentage of mitotically arrested cells was also confirmed by flow cytometry after PFKFB4 depletion and 50 nM paclitaxel in SKOV3 cells and **D**. OC 316 cells. Data are expressed as the mean +SEM of three experiments.

PFKFB4 belongs to a family of bifunctional enzymes, known as PFKFB1-4, which have an important role in regulating glycolysis [32] (Figure 3C). The phosphofructokinase domain of PFKFB enzymes produces fructose 6-phosphate, while their phosphatase activity yields fructose 2,6-bisphosphate [33]. Fructose 2,6-bisphosphate is an allosteric activator of phosphofructokinase 1, which controls one of the critical rate-limiting steps of glycolysis [34, 35]. siRNA-mediated depletion of *PFKFB4* significantly decreased the number of mitotically arrested SKOV3 cells following paclitaxel treatment, but not during spontaneous mitosis. This suggested that PFKFB4 depletion was detrimental only to SKOV3 cells undergoing prolonged mitotic arrest. To validate the screen results we used pooled siRNA duplexes to deplete PFKFB4 prior to paclitaxel treatment (Figure 4A) and quantified the number of mitotic cells present compared to the non-targeting control. Paclitaxel treatment of PFKFB4 depleted SKOV3 cells resulted in a significant decrease in the mitotic cell index compared to the non-targeting control when analysed by both immunofluorescence (Figure 4B) *p*<0.0001, two-way ANOVA multiple comparison) and flow cytometry (Figure 4C, *p*<0.0001, two-way ANOVA multiple comparison). To exclude off-target effects, we used four individual siRNA duplexes to deplete PFKFB4 (Supplementary Figure 2A), all of which decreased the mitotic cell index when quantified by immunofluorescence (Supplementary Figure 2B). PFKFB4 depletion was also investigated in OC 316 cells, which exhibited a reduction in AKT activity and ATP levels during mitotic arrest by paclitaxel, and a significant reduction in mitotic cell number was also identified (Figure 4D, *p=*0.006, two-way ANOVA multiple comparison). In addition to paclitaxel, preliminary data investigating PFKFB4 depletion in SKOV3 cells prior to mitotic arrest using 0.5μM nocodazole decreased the number of mitotic cells to a similar extent as what is observed following paclitaxel treatment (data not shown).

**Figure 5:**
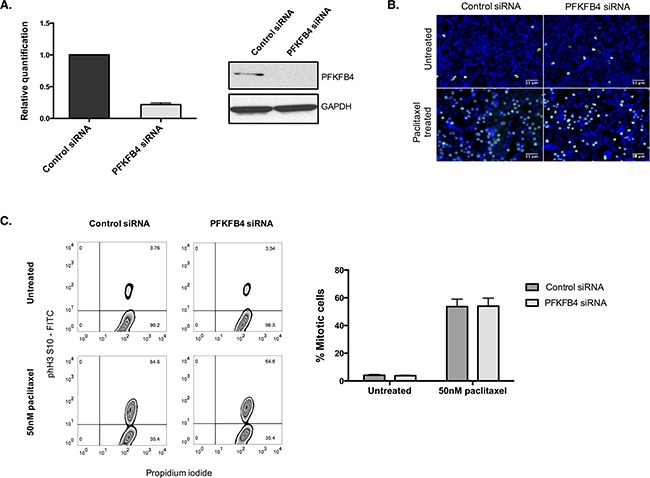
PFKFB4 depletion in A2780 cells has no effect on the number of mitotically arrested cells **A**. *PFKFB4* mRNA and protein levels were assessed by qPCR and western blotting after PFKFB4 depletion using pooled siRNA for 72 h. **B**. After siRNA-mediated depletion of *PFKFB4* for 72 h followed by 50 nM paclitaxel for 16 h, cells were fixed and stained for immunofluorescence. Images are shown with Hoechst stain (blue) and phospho-histone H3 (S10) (green). **C**. The percentage of mitotically arrested cells was confirmed by flow cytometry after PFKFB4 depletion and 50 nM paclitaxel. Data are expressed as the mean +SEM of three experiments.

To determine whether the effect of PFKFB4 depletion on the number of mitotically arrested cells is due to the vulnerability that they possess during mitotic arrest, this analysis was repeated in the A2780 cell line and siRNA-mediated depletion of *PFKFB4* was achieved (Figure 5A). There was no significant effect of PFKFB4 depletion on the number of mitotically arrested cells when using immunofluorescence staining (Figure 5B). Analysis using flow cytometry confirmed the maintenance of mitotically arrested cell number after PFKFB4 depletion (Figure 5C) two-way ANOVA multiple comparison). Therefore, we concluded that SKOV3 cells are vulnerable to PFKFB4 depletion during mitotic arrest, whereas A2780 are resistant.

### PFKFB4 depletion can increase mitotic cell death in paclitaxel treated ovarian cancer cells

**Figure 6:**
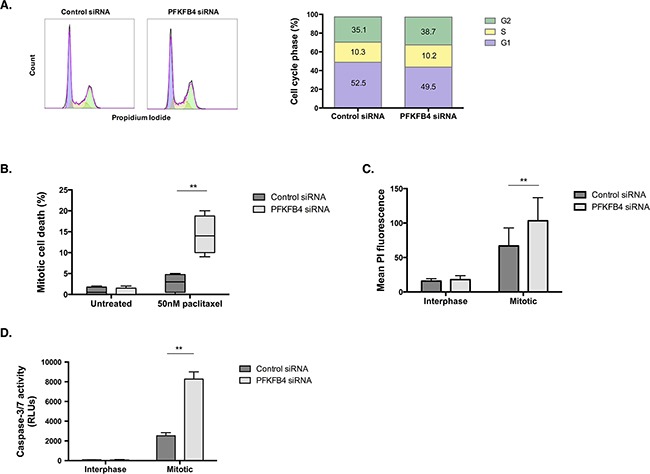
PFKFB4 depletion increases death of mitotically arrested SKOV3 cells **A**. Cell cycle analysis after siRNA-mediated depletion of *PFKFB4* for 72 h was assessed by propidium iodide staining and flow cytometry. **B**. Mitotic cell death was assessed using time-lapse microscopy for 16 h and following the fate of over 200 mitotically arrested cells in each condition. **C**. After PFKFB4 depletion and mitotic cell isolation, the incorporation of propidium iodide was assessed by flow cytrometry and **D**. the activity of caspases 3 and 7 was measured using the Caspase-Glo 3/7 assay. Data are expressed as the mean +SEM of three experiments.

The effect of PFKFB4 depletion on reducing the number of mitotically arrested cells after paclitaxel treatment could occur as a result of inducing mitotic cell death, arresting cell cycle progression, or increasing mitotic slippage, or a combination of the three. Depletion of PFKFB4 did not significantly affect cell cycle progression in SKOV3 cells when using propidium iodide staining and flow cytometry (Figure 6A). Time-lapse microscopy was performed to follow the fate of mitotic cells for 24 h after PFKFB4 depletion and paclitaxel treatment as we have previously described [3]. We observed a significant increase in mitotic cell death following PFKFB4 depletion compared to the non-targeting control (Figure 6B, *p=*0.0056, two-way ANOVA multiple comparison), indicating that PFKFB4 is important for ovarian cancer cell survival during prolonged mitosis. In support of this, a significant increase in PI staining was observed in mitotically arrested cells after PFKFB4 depletion when compared to the non-targeting control (Figure 6C, *p=*0.0049, two-way ANOVA multiple comparison). Moreover, the activity of caspases 3 and 7 were significantly elevated in mitotically arrested cells after PFKFB4 depletion compared to the non-targeting control (Figure 6D, *p=*0.0016, two-way ANOVA multiple comparison). The increased activity of caspases 3 and 7 suggests that PFKFB4 depletion may induce apoptosis during prolonged mitosis [36], thus decreasing the mitotic index.

**Figure 7:**
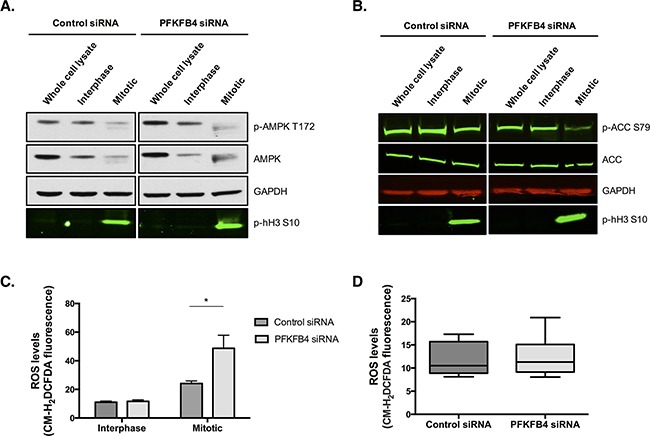
PFKFB4 depletion increases reactive oxygen species in mitotically arrested SKOV3 cells without increasing AMPK activity After control siRNA or siRNA-mediated depletion of *PFKFB4* for 72 h, cells were treated with 50 nM paclitaxel for 16 h and separated by mitotic shake-off into the mitotic and interphase populations. **A**. Western blotting was used to determine the phosphorylation of AMPK at T172 and **B**. ACC at S79. **C**. Levels of ROS were determined using CM-H2DCFDA staining and flow cytometry after siRNA-mediated depletion of PFKFB4 in the interphase and mitotically arrested populations separated by mitotic shake-off and **D**. in the whole population without paclitaxel treatment. Data are expressed as the mean +SEM of three experiments.

Taking into account the critical role that PFKFB4 has in regulating glycolysis via the production of fructose 2,6-bisphosphate [35, 37, 38], and the apparent decrease in ATP levels we have observed during mitotic arrest, it could be hypothesised that the increase in mitotic cell death after PFKFB4 depletion is due to a further critical decrease in ATP levels. Substantial decreases in ATP concentration have been shown to trigger apoptosis [39–41]. However, AMPK phosphorylation at T172, an activating site within the T-loop, suggests that mitotically arrested cells are not energetically stressed after PFKFB4 depletion because AMPK phosphorylation and expression were reduced compared to interphase cells (Figure 7A). The lack of an increase in AMPK activity in mitotically arrested SKOV3 cells was surprising given that AMPK activity has previously been found to increase in mitotically arrested cells [42]. However, the study by Domenech *et al*., did this, the or arrest cells by paclitaxel treatment alone. In support of this, the phosphorylation of the key enzyme in fatty acid synthesis, acetyl-CoA carboxylase (ACC), which is phosphorylated and inactivated by AMPK [43], was reduced in the mitotically arrested population after PFKFB4 depletion (Figure 7B). AMPK phosphorylation was also reduced in the mitotically arrested population treated with non-targeting siRNA, indicating that these cells are not energetically stressed, despite the reduction in ATP levels. Taken together, these data suggest that mitotically arrested cells are not energetically stressed and that the increase in mitotic cell death after PFKFB4 depletion and paclitaxel treatment is not due to a critical decrease in ATP levels and subsequent energetic stress.

As ovarian cancer cells do not appear to be energetically stressed after PFKFB4 depletion, we hypothesised that the levels of reactive oxygen species (ROS) may be increased after PFKFB4 depletion. We measured the levels of ROS in mitotic and interphase SKOV3 cells after PFKFB4 depletion followed by paclitaxel treatment and found the levels of ROS to be significantly increased in mitotically arrested cells after PFKFB4 depletion compared to the non-targeting control (Figure 7C, *p=*0.0124, two-way ANOVA multiple comparison). These data suggest that the increase in ROS occurs specifically during prolonged mitotic arrest and not during interphase. In support of this, no significant difference in the levels of ROS was found after PFKFB4 depletion compared to the non-targeting control in untreated cells (Figure 7D).

### The PFKFB pathway is important during mitotic arrest in ovarian cancer cells

**Figure 8:**
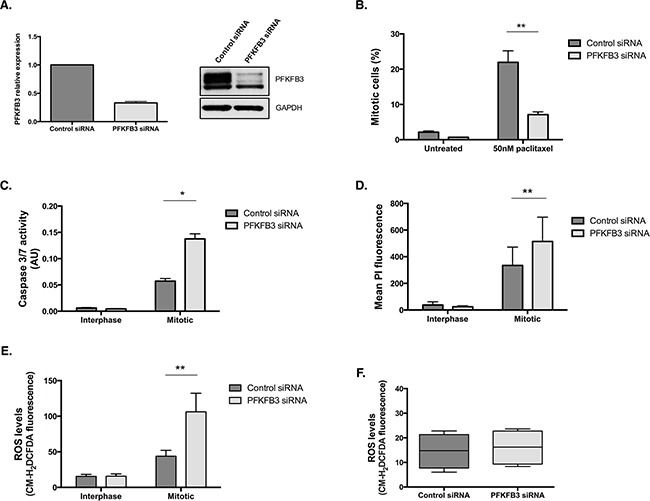
PFKFB3 is important for cell survival during paclitaxel-induced mitotic arrest **A**. *PFKFB3* mRNA and protein expression after siRNA-mediated depletion of *PFKFB3* for 72 h was determined by qPCR and western blotting. **B**. The quantification of mitotically arrested cells by flow cytometry, **C**. caspase 3/7 activity using Caspase-Glo 3/7 assay **D**. propidium iodide incorporation by flow cytometry and **E**. ROS levels using CM-H2DCFDA staining and flow cytometry were assessed after siRNA-mediated depletion of *PFKFB3* for 72 h followed by 50 nM paclitaxel for 16 h and a mitotic shake-off in SKOV3 cells. **F**. The levels of ROS after PFKFB3 depletion alone, without paclitaxel treatment or mitotic cell isolation, was also analysed using CM-H2DCFDA staining and flow cytometry. Data are expressed as +SEM of three experiments.

Of all the PFKFB family members, PFKFB3 has been the most intensively studied and is overexpressed in many human cancers, including ovarian cancer [44]. To investigate the importance of the PFKFB family during mitotic arrest, we examined the effects of siRNA-targeted depletion of *PFKFB3* on paclitaxel treated ovarian cancer cells (Figure 8A). Similar to PFKFB4 targeting, depletion of PFKFB3 caused a significant decrease in mitotic cell number after paclitaxel treatment compared to the non-targeting control (Figure 8B, *p=*0.006, two-way ANOVA multiple comparison). The decrease in mitotic index was accompanied by a significant increase in caspase 3 and 7 activity in mitotically arrested cells depleted of PFKFB3 (Figure 8C, *p=*0.0165, two-way ANOVA multiple comparison). There was also a significant increase in the levels of PI staining in mitotically arrested cells after PFKFB3 targeting compared to the non-targeting control (Figure 8D, *p=*0.0022, two-way ANOVA multiple comparison). The increase in caspase 3 and caspase 7 activity and the greater PI incorporation both suggest that there is an increase in cell death when mitotically arrested cells are depleted of PFKFB3. In addition to this, ROS levels displayed a significant increase specifically in mitotically arrested cells depleted of PFKFB3 (Figure 8E, *p=*0.0051, two-way ANOVA multiple comparison). An increase in ROS after PFKFB3 depletion has previously been noted by Klarer *et al*., 2014 [45], however this was not observed specifically in mitotically arrested cells as it is in this research. No increase in ROS was observed after siRNA-mediated depletion of PFKFB3 alone (Figure 8F). This may demonstrate tissue-specific regulation of these enzymes or highlight a previously unrecognised role during mitotic arrest.

*PFKFB3* depletion was achieved using stealth siRNA duplexes transfected with RNAiMAX, whereas in the siRNA screen, Dharmacon siRNA duplexes were used and transfected with DharmaFECT4 reagent. The latter approach has not been employed by any other studies depleting PFKFB3 using siRNA duplexes [42, 45–47], indicating that this technique may not be effective at depleting *PFKFB3*, which would explain why PFKFB3 was not identified in the kinome screen.

### Restoration of AKT activity in mitotically arrested SKOV3 cells does not confer resistance to PFKFB4 depletion

**Figure 9:**
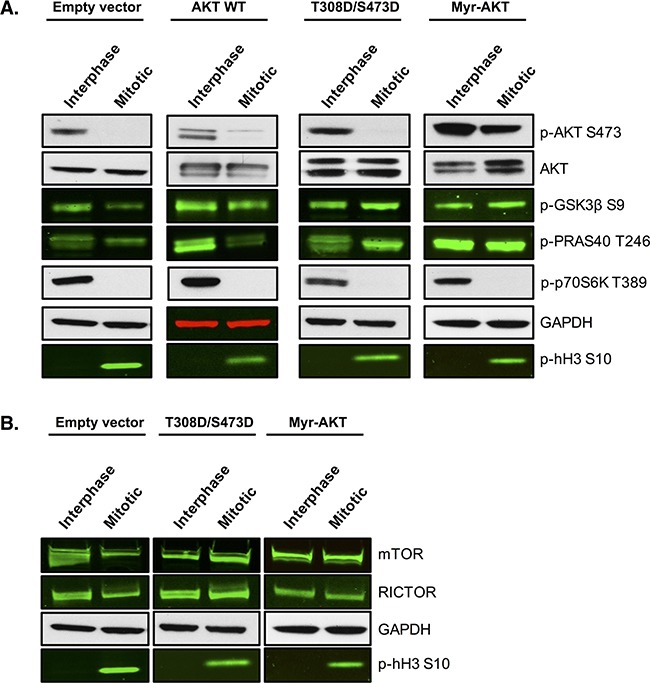
Overexpression of AKT restores the phosphorylation of direct AKT substrates SKOV3 cell lines stably overexpressing wild-type AKT, T308D/S473D or Myristoylated-AKT were generated using lentiviral transfection of HEK 293T cells and transduction in SKOV3 cells. **A**. Western blotting measured the phosphorylation status of AKT and its downstream targets and **B**. the expression of proteins upstream of AKT in the empty vector, wild-type AKT, T308D/S473D and Myristoylated-AKT cell lines. In the western blots, GAPDH is the loading control, phospho-histone H3 S10 is the mitotic marker and images represent one of three experiments.

We next tested whether maintenance of AKT signalling during mitotic arrest could render cells resistant to PFKFB4 targeting. To address this, we produced SKOV3 cell lines stably expressing constitutively active AKT through two different mechanisms; either by the addition of two phosphomimetic mutations T308D and S473D or through the myristoylation of the N-terminus of AKT (Myr-AKT). Both of these alterations have previously been shown to lead to constitutive activation of AKT [48, 49] and to directly regulate GLUT4 translocation to the plasma membrane [50, 51]. To investigate whether AKT activity was restored during mitotic arrest, we first analysed the phosphorylation of AKT and its downstream targets (Figure 9A). The phosphorylation of two direct substrates of AKT, PRAS40 and GSK3β, was increased compared to the empty vector control and the wild-type AKT cell lines (AKT WT). In addition, maintenance of AKT phosphorylation at S473 in mitotically arrested cells was observed in Myr-AKT. Moreover, protein levels of mTOR and RICTOR were restored in both the T308D/S473D and Myr-AKT cell lines (Figure 9B), suggesting that there may be a positive feedback loop upstream causing further activation of the pathway. Intriguingly, the phosphorylation of p70S6K was not rescued in any of the cell lines stably expressing AKT, which suggests that either AKT activity is not fully restored during mitotic arrest, or as p70S6K is not a known direct substrate of AKT, additional components that are required for its phosphorylation are not functional in these cell lines.

**Figure 10:**
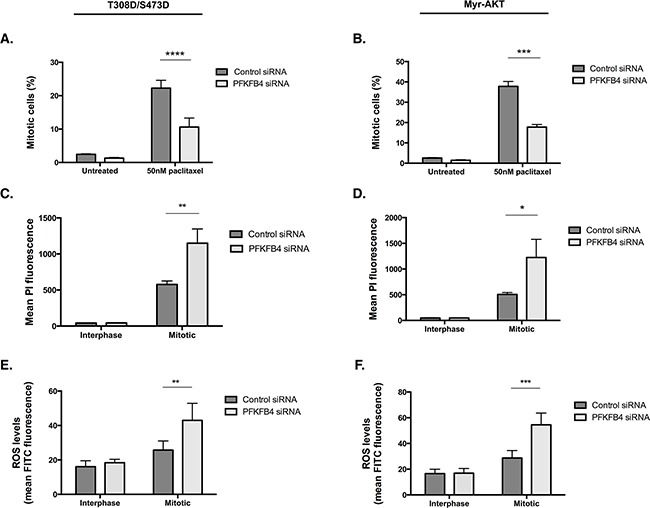
Overexpression of constitutively active AKT does not confer resistance to PFKFB4 depletion **A, B**. After siRNA-mediated depletion of *PFKFB4* for 72 h, 50 nM paclitaxel for 16 h and mitotic cell isolation, the percentage of mitotically arrested cells was determine using flow cytometry, **C, D**. PI incorporation was measured by flow cytometry and **E, F**. levels of ROS were measured using CM-H2DCFDA staining and flow cytometry in T308D/S473D and Myr-AKT cell lines. Data are expressed as the mean +SEM of three experiments.

Given that we observed increased activity of the AKT pathway directly upstream and downstream of AKT in the T308D/S473D and Myr-AKT cell lines, we investigated whether there was any rescue in the levels of ROS or mitotic cell index after PFKFB4 depletion. There was still at least a 50% reduction in the number of mitotically arrested cells after PFKFB4 compared to the non-targeting control in both the T308D/S473D (Figure 10A, *p*<0.0001, two-way ANOVA multiple comparison) and Myr-AKT (Figure 10B, *p=*0.0004, two-way ANOVA multiple comparison) cell lines. This was accompanied by a significant increase in PI incorporation into mitotically arrested cells depleted of PFKFB4 in T308D/S473D (Figure 10C, *p=*0.0046, two-way ANOVA multiple comparison) and Myr-AKT (Figure 10D, *p=*0.0426, two-way ANOVA multiple comparison), suggesting that there is an increase in cell death. Levels of ROS in both T308D/S473D (Figure 10E, *p=*0.0069, two-way ANOVA multiple comparison) and Myr-AKT (Figure 10F, *p=*0.0.0003, two-way ANOVA multiple comparison) were also significantly increased in mitotically arrested cells after PFKFB4 depletion compared to the non-targeting control. The effects of individual siRNA duplexes were tested to check for off-target effects, however all of them displayed a significant decrease in mitotically arrested cells after PFKFB4 depletion (Supplementary Figure 3A-3D). These data indicate that partial restoration of AKT signalling during mitotic arrest does not protect cells from the detrimental effects of PFKFB4 depletion. Moreover, it may indicate that PFKFB4 depletion is not directly exploiting the vulnerability created from the loss of AKT activity during mitotic arrest and is producing an effect through an alternative pathway.

## DISCUSSION

This work has uncovered a novel function of the enzymes PFKFB3 and PFKFB4 in ovarian cancer cells during mitotic arrest. Depletion of PFKFB3 and PFKFB4 prior to paclitaxel treatment caused a significant reduction in mitotic cell number, an increase in ROS and PI incorporation, and enhanced caspase 3/7 activity in mitotically arrested cells. While numerous studies have identified the importance of PFKFB3 and PFKFB4 in cancer cell survival [45–47], we are the first to investigate the impact of their depletion in ovarian cancer cells and identify their importance during mitotic arrest.

Another important aspect of this work is that it has highlighted a targetable vulnerability in ovarian cancer cells during mitotic arrest and possibly during spontaneous mitosis, due to the reduction in AKT signalling and glucose 6-phosphate, and a significant decrease in ATP levels. This is intriguing given that the ovarian cancer cell lines used in this study possess a *PI3KCA* mutation [20–22] causing constitutive activation of the PI3K/AKT pathway, which indicates the importance of this pathway and the potential vulnerability caused by its temporary inactivity during mitosis. The observation that the A2780 cell line maintains AKT activity during mitotic arrest indicates that the loss of AKT is not simply because the cells are asynchronous. It would be informative to investigate PFKFB4 depletion in *PTEN* null cells to determine whether they are also unaffected following PFKFB4 depletion during mitotic arrest. Moreover, the fact that A2780 cells remain unaffected by PFKFB4 depletion demonstrates the specificity of this vulnerability and that it is not universal across all ovarian cancer cell lines. Additionally, whether PFKFB4 depletion is exploiting this specific vulnerability is unclear as the constitutive activation of AKT did not rescue the observed increase in mitotic cell death. An interaction between AKT signalling and PFKFB4 was also recently observed by Pegoraro *et al*., 2015 [52], where they found that PFKFB4 depletion influenced AKT phosphorylation, but no direct link was established between the two proteins. It could be that the loss of AKT phosphorylation following PFKFB4 depletion makes cells more vulnerable to AKT inhibition, however this has not been determined in this work.

Our high content siRNA screen successfully identified PFKFB4 as an important regulator of ovarian cancer cell survival following mitotic arrest by paclitaxel. These results are supported by two independent groups that have conducted unbiased screens for genes essential for cancer survival and found that PFKFB4 is required for both glioma stem-like cell [53] and prostate cancer cell survival [47], but not for normal cell survival. However, this is the first study to identify an increase in ROS specifically in mitotically arrested cells after PFKFB3 or PFKFB4 depletion. We predict that this specific increase in ROS only in mitotically arrested cells is due to a decrease in mitochondrial membrane potential [54] or a suppression of glycosylation [55]. An increase in ROS levels after depletion of PFKFB3 and PFKFB4 has been observed in a number of other studies [45, 47, 56], however in the work presented here an increase in ROS was observed only in the mitotically arrested cells after PFKFB3 or PFKFB4 depletion and not in interphase cells. This discrepancy might be due to PFKFB3 and PFKFB4 having distinct roles in ovarian cancer cells. The main function of PFKFB4 in cancer cells has been an area of debate, with a number of studies indicating a predominant phosphatase activity [47, 56] and others suggesting a dominant kinase activity [57, 58].

It is unlikely that the increase in mitotic cell death is attributable to oxidative stress as treatment with the antioxidant N-acetyl cysteine could not rescue the decrease in mitotic cell number despite reducing the levels of ROS (data not shown). The production of ROS may therefore be a by-product and not the causal factor in mitotic cell death following PFKFB4 depletion. Mitotic cell death has previously been attributed to the energetic capacity of cells during mitotic arrest [42]. Mitotic arrest can increase mitophagy, which reduces oxidative respiration and ATP, forcing the cell to rely more heavily on glycolytic metabolism. This places a strong requirement on processes that promote glycolysis, such as AMPK-mediated PFKFB3 activation [42]. However, in this work, AMPK was not phosphorylated after PFKFB3 depletion in mitotically arrested cells, which suggests that they are not energetically stressed and an alternative cause of mitotic cell death is most likely.

The panel of cell lines analysed in this work represent some of the most commonly used cell lines in ovarian cancer research, for example SKOV3 cells and A2780 cells, which account for approximately 60% of publications related to ovarian cancer [21]. In addition, we have confirmed that the SKOV3 cell line used in this work possess a *TP53* mutation, which is a hallmark of high grade serous ovarian cancer (HGSOC). However, to determine the applicability of this work to HGSOC and not simply epithelial ovarian cancer in general, cell lines that more accurately represent this subtype should be used, such as those developed by Kreuzinger *et al*., 2015 [59]. A further limitation of this work is the nature and duration of mitotic arrest by paclitaxel. Cells were arrested for 16 hours in cell culture medium containing 50 nM paclitaxel, which may not accurately represent the pharmacokinetics of paclitaxel or the nature of mitotic arrest *in vivo*. Paclitaxel is known to undergo triphasic elimination in the plasma with the C_max_ being approximately 4 μmol/L following the standard three-weekly dose of 175 mg/m^2^/h [60]. However, there is a lack of information regarding the exact concentration of paclitaxel that reaches the tumour, so although this work provides a useful insight into targeting PFKFB4 in mitotically arrested ovarian cancer cells, whether the nature of the mitotic arrest following paclitaxel treatment reflects that of an *in vivo* environment has yet to be established.

The main objectives of this study were to identify targetable vulnerabilities in ovarian cancer cells during mitotic arrest and to determine whether we can exploit these for therapeutic benefit. In summary, we have identified a metabolic vulnerability in the majority of ovarian cancer cells during mitotic arrest arising from a striking decrease in AKT activity, a reduction in glucose 6-phosphate, and a significant decrease in ATP levels. Moreover, we have demonstrated that the depletion of either PFKFB3 or PFKFB4 can significantly increase cell death in mitotically arrested cells. This work has identified a previously unrecognised role of PFKFB4 in mitotically arrested ovarian cancer cell survival and supports the notion that combining mitotic-targeted therapies with glycolytic inhibitors may potentiate the effects of antimitotics in ovarian cancer.

## MATERIALS AND METHODS

### Cell lines and cell culture

SKOV3, OVCAR-3, OC 316, UPN-251 and HEK-293T cell lines were obtained from the American Tissue Type Culture Collection (ATCC), A2780 cells were purchased from Sigma-Aldrich. SKOV3 cells were maintained in McCoy's 5A medium (Thermo Fisher Scientific), A2780 and OVCAR-3 cells were cultured in RPMI 1640 medium (Thermo Fisher Scientific), while HEK293T, OC 316, UPN-251 were cultured in DMEM (Dulbecco's modified Eagle's medium) (Thermo Fisher Scientific). Growth medium was supplemented with 10% (v/v) fetal bovine serum (Thermo Fisher Scientific) and 1% (v/v) penicillin/streptomycin (Thermo Fisher Scientific) and cells were grown at 37**°**C and 5% CO_2_. SKOV3 cell lines stably expressing AKT1 or AKT1 mutants were grown in media containing 50ng/l puromycin (Sigma-Aldrich). Stock solutions of 1 mM Paclitaxel (Sigma-Aldrich) were prepared with dimethyl sulphide (DMSO) (Sigma-Aldrich).

### Mitotic cell isolation

Cells were treated with 50 nM paclitaxel for 16 h, after which media was removed and cells were washed once with PBS. Fresh PBS was added to the flask and mitotic cells were detached by gentle shaking. Detachment of cells was assessed by viewing the cells under the AE2000 Inverted microscope (Motic) and shaking continued until the majority of mitotic cells had detached. Spontaneous mitotic cells were obtained using the same protocol, however, cells were not treated with paclitaxel and therefore eight T175 flasks were used to obtain the small number of spontaneous mitotic cells. Remaining interphase cells were collected by trypsinisation. Validation of efficient isolation of mitotic cells was always assessed by the presence of phospho-histone H3 S10 staining in the mitotic population and not the interphase cells.

### SDS PAGE and western blotting

Samples were prepared for SDS-PAGE by mixing 30 μg of protein with 1x NuPage LDS Sample Buffer (Thermo Fisher Scientific) and deionised water before heating at 95**°**C for 10 min. For analysis of proteins larger than 200kDa, 1x NuPAGE Reducing Agent was added to the samples prior to boiling. Samples were then subjected to SDS-PAGE using 1.5mm NuPage 4-12% Bis-Tris precast polyacrylamide gels (Thermo Fisher Scientific) with 1x NuPage MOPs Running Buffer diluted in deionised water (Thermo Fisher Scientific) or 1.5mm NuPage 3-8% Tris-Acetate precast polyacrylamide gels (Thermo Fisher Scientific) with 1x NuPage Tris-Acetate Running Buffer diluted in deionised water (Thermo Fisher Scientific) supplemented with 500 μl NuPage Antioxidant in the inner buffer chamber for proteins over 200kDA. SeeBlue Plus 2 Pre-stained protein standard (Thermo Fisher Scientific) was loaded into the first lane of each gel followed by the set of samples and gels were run at 160V for approximately 50 min. Proteins were transferred to a nitrocellulose membrane using the iBlot dry gel transfer device (Thermo Fisher Scientific). Membranes were incubated in blocking buffer (5% skimmed milk powder (Sigma-Aldrich diluted in TBST (1x Tris-buffered saline and 0.1% Tween-20)) at room temperature for 1 h. Primary antibodies were diluted in blocking buffer and incubated with the membrane overnight at 4°C. Membranes were washed three times for 15 min in TBST before incubating with either horseradish peroxidase(HRP)-conjugated secondary antibodies (Goat anti-rabbit and Goat anti-Mouse, Active Motif) or LI-COR Odyssey secondary antibodies (Goat anti-Mouse IRDye 680RD and Goat anti-rabbit IRDye 800CW) for 1 h at room temperature. Membranes were washed three times for 15 min in TBST and visualised using Pierce enhanced chemiluminescence Western Blotting Substrate (Thermo Fisher) for HRP-conjugated secondary antibodies or the Odyssey Imager (LI-COR) for fluorescent secondary antibodies.

### RNA extraction and quantitative PCR

Total RNA was isolated using RNeasy mini kit (Qiagen) including the DNase-1 treatment according to the manufacturer's protocol and quantified using the NanoDrop ND-1000 spectrophotometer (Nanodrop Technologies, Inc). 1 μg of RNA was reverse transcribed using TaqMan® Reverse Transcription Kit (Applied Biosystems). Quantitative real-time PCR was performed using SYBR® Green PCR Master Mix (Applied Biosystems) in a 20 μl reaction using a MicroAmp Fast Optical 96-Well Reaction Plate according to the manufacturer's protocol on the 7500 Fast Real-Time PCR System (Applied Biosystems). The specificity of the PCR products were confirmed by melting curve analysis. cDNA amplification was normalised against *GAPDH* and *ACTB*. Expression ratios relative to the control sample were calculated as previously described [61].

### Transfections

Transient protein depletion experiments with siRNA duplexes purchased from GE Healthcare were conducted using a reverse transfection method with siRNA at a final concentration of 40 nM. 4 μl siRNA specific to the gene of interest, non-targeting siRNA or Dharmafect 4 transfection reagent (Dharmacon, GE Healthcare) were diluted separately in 200 μl Opti-MEM for 5 min according to the manufacturers protocol. 200 μl DharmaFECT 4-Opti-MEM complex was combined with 200 μl siRNA-Opti-MEM complex and incubated for 20 min prior to aliquoting the full 400 μl Dharmafect 4-siRNA complex into each well of a 6-well plate. Cells were resuspended in media and diluted to 90,000 cells/ml for SKOV3 and OC 316 cells, and 110,000 cells/ml for A2780. 1.6 ml of the diluted cells were added on top of the DharmaFECT 4-siRNA complex and plates were left for 72 h at 37°C and 5% CO_2_ to achieve efficient protein depletion. For T25 flasks, 6 μl siRNA or DharmaFECT4 were diluted in 300 μl Opti-MEM and 2.4 ml cells were added on top of 600 μl DharmaFECT 4-siRNA complex.

### High-content siRNA kinome screen

SKOV3 cells were plated in a 384-well format and treated with Dharmacon siRNA kinome library targeting 779 different genes for 48 h. Cells were treated with 10 nM paclitaxel or vehicle control for 24 h prior to fixation with 4% paraformaldehyde for 5 min. Cells were permeabilised with ice-cold absolute ethanol and left overnight at −20°C prior to adding wash buffer (Tris-buffered saline, 0.2% Triton X-100 and 0.04% SDS) for 10 min. Cells were blocked in blocking buffer (1.5% bovine serum albumin diluted in Tris-buffered saline) for 1 h at room temperature. Cells were incubated with anti-phospho-histone H3 S10 antibody diluted in blocking buffer for 1 h at room temperature. Three washes were performed with wash buffer for 5 min each and Alexa Fluor 488 donkey anti-rabbit was diluted in blocking buffer and added to each well for 1 h at room temperature in the dark. Cells were washed three times as before and incubated with 10 μg/ml Hoechst 33342 (Thermo Fisher Scientific) diluted in PBS for 10 min at room temperature. The Hoechst stain was removed and PBS was added to each well prior to image acquisition using the In Cell Analyzer 1000 (GE Healthcare) as previously described [62]. Data analysis was carried out using R programming language and software. Cells were assigned to specific wells and the mean pixel intensity values were calculated for each Hoechst and phospho-histone H3 S10 cell and background values were subtracted. The percentage of mitotic cells was calculated for each well by dividing the number of cells staining positive for phospho-histone H3 S10 by the total number of cells that stained positive for Hoechst. The median of triplicate values was obtained for each condition.

### Immunofluorescence

Cells were grown to 80% confluence on cover slips in 12-well plates prior to fixation with 0.5 ml 4% methanol-free formaldehyde (Thermo Fisher Scientific) for 4 min at room temperature. Permeabilisation was achieved using 0.5 ml 100% ice-cold ethanol and incubation at −20°C overnight. Ethanol was removed and 1 ml wash buffer (1x TBS, 0.2% Triton X100 and 0.04% SDS, Sigma-Aldrich was added to the wells for 5 min. Cells were blocked in 1 ml blocking buffer (3% bovine serum albumin diluted in 1xTBS) for 1 h at room temperature. Cover slips were incubated with 60 μl primary antibody diluted in blocking buffer for 1 h at room temperature. Three washes were performed with 1 ml wash buffer for 5 min each. Secondary antibodies (Alexa Fluor 488 donkey anti-rabbit or Alexa Fluor 568 donkey anti-mouse, Thermo Fisher Scientific) were diluted in blocking buffer and 60 μl was added to each cover slip for 1 h at room temperature in the dark. Cells were washed as before prior to mounting the coverslips with mounting medium containing DAPI (Vectashield) on microscope slides (ThermoScientific) sealed with nail varnish at the edges. Slides were stored at 4°C in the dark until analysis. Images were analysed on the Zeiss Observer Z1 Microscope using a 40X oil objective or a 20X objective. Images were analysed using ImageJ software.

### Quantification of mitotic cells by flow cytometry

Cells were centrifuged at 1500 rpm (365 xg) for 5 min to pellet the cells. Supernatants were removed and pellets were resuspended thoroughly in 0.5 ml PBS and transferred to Falcon 5 ml Round Bottom flow cytometry tubes (Scientific Laboratory Supplies). 0.5 ml 8% methanol-free formaldehyde (Thermo Fisher Scientific) was added to give a final concentration of 4% and incubated for 6 min at room temperature. Fixative was removed by centrifugation at 1500 rpm (365 xg) for 5 min and samples were thoroughly resuspended in 1 ml ice-cold 100% ethanol and placed at −20°C overnight to allow cell permeabilisation. Cells centrifugation at 5000 rpm for 5 min and washed in 1 ml blocking buffer (3% bovine serum albumin in 1xTBS). Cells were collected by centrifugation as described above and resuspended in 1 ml blocking buffer for 1 h at room temperature. Cells were pelleted again and incubated with rabbit anti-phospho-histone H3 S10 diluted in blocking buffer for 1 h at room temperature. Cells were washed twice in 1 ml blocking buffer before incubation with the secondary antibody (Alexa Fluor 488 donkey anti-rabbit) diluted in blocking buffer for 1 h at room temperature in the dark. Cells were pelleted by centrifugation at 5000 rpm for 5 min and washed in 1 ml blocking buffer. Cells were finally resuspended in 0.5 ml 3 μM Propidium Iodide solution (PI) (Thermo Fisher Scientific) diluted in PBS to exclude dead cells as PI is membrane impermeant and analysed on the CyAn ADP Analyser (Beckman Coulter). Data from approximately 100,000 cells from each sample were analysed using FlowJo software.

### Cell cycle analysis

Cell cycle analysis was performed using flow cytometry and propidium iodide (PI) staining. After incubation with PFKFB4 siRNA for 72 h, cells were harvested into Falcon 5 ml Round Bottom flow cytometry tubes (BD Falcon) prior to centrifugation at 1500 rpm (365 xg) for 5 min. Cells were resuspended in 1 ml PBS with 3% FBS and centrifuged again at 1500 rpm (365 xg) for 5 min. 1 ml ice-cold 70% ethanol (Sigma-Aldrich was added drop-wise to each sample prior to vortexing and centrifugation at 3000 rpm for 4 min. Cells were washed in 1 ml PBS with 3% FBS and pelleted again by centrifugation at 3000 rpm for 4 min. 0.5 ml 100 μg/ml RNase A (Thermo Fisher Scientific) and 40 μg/ml PI diluted in PBS with 3% FBS was added to each sample and incubated at 37°C and 5% CO_2_ for 40 min. Samples were collected by centrifugation at 3000 rpm for 4 min and resuspended in 1 ml PBS with 3% FBS for analysis on CyAn ADP Analyser (Beckman Coulter). Cells were kept covered prior to analysis. PI was analysed on a linear scale with Area vs Peak to gate out doublets and analysed at a low flow rate (<400 events/second).

### Detection of reactive oxygen species

Reactive oxygen species (ROS) levels were measured using the cell permeable indicator CM-H_2_DCFDA (Thermo Fisher Scientific). Cells were incubated with PFKFB4 siRNA or PFKFB3 siRNA for 72 h in T25 flasks. After 72 h, 2 ml 50 nM paclitaxel or DMSO control (Sigma-Aldrich) diluted in media were added to cells and incubated overnight at 37°C and 5% CO_2_. The next day, mitotic cells were separated from interphase cells and placed in Falcon 5 ml Round Bottom flow cytometry tubes (Scientific Laboratory Supplies) prior to centrifugation at 1500 rpm (365 xg) for 5 min. CM-H_2_DCFDA was freshly reconstituted in DMSO and cells were resuspended in 1 ml 3 μM CM-H_2_DCFDA diluted in warm McCoy's 5A media and incubated for 30 min at 37°C and 5% CO_2_. 1 ml 1 mM Hydrogen peroxide (H_2_0_2_) (Sigma-Aldrich) and 3 μM CM-H_2_DCFDA diluted in warm McCoy's 5A media were added to cells as a positive control. After 30 min, cells were centrifuged at 1500 rpm (365 xg) for 5 min and resuspended in 0.5 ml ice-cold 10 μg/ml propidium iodide (Thermo Fisher Scientific) (535nm/617nm) diluted in PBS. Samples were placed on ice and kept in the dark until analysis on CyAn ADP Analyser (Beckman Coulter). Samples were also tested by western blot for the presence of phospho-histone H3 S10 in the mitotic samples.

### Caspase 3/7 measurements

The Caspase-Glo 3/7 Assay (Promega) was performed following the manufacturer's protocol. Caspase-Glo 3/7 Buffer and Caspase-Glo 3/7 Substrate were equilibrated to room temperature prior to use. Caspase-Glo 3/7 substrate was reconstituted by transferring the entire Caspase-Glo 3/7 Buffer into the bottle. Cell pellets were resuspended in media and counted as previously described. Cells were diluted to 100,000 cells/ml and 50 μl of cell suspension was plated in an opaque-walled 96-well plate in triplicate. Media alone was added in triplicate to allow the exclusion of background luminescence and Staurosporine (Cell Signaling Technology) treatment was used as a positive control (0.3 μM for 16 h). 50 μl of the reconstituted substrate was added to each well in the dark and the plate was covered in foil and placed on a plate shaker for 30 seconds prior to incubation at room temperature for 1 h. The plate was read on a luminescent plate reader. Triplicate measurements were averaged and the culture media background fluorescence was subtracted from the average of each experimental sample.

### Time-lapse microscopy

Transfections with siRNA were set up as previously described, but scaled down for use of a 12-well plate, meaning 800 μl cell suspension at the densities stated were combined with 200 μl DharmaFECT 4-siRNA complex per well. After transfection for 72 h, cells were treated with 50 nM paclitaxel or DMSO control and visualised by time-lapse microscopy using a 20X objective on the Leica DMIRE2 Hamamatsu ORCA-ER using HCI Image acquisition software while keeping the cells at 37°C and 5% CO_2_. Five fields of view in each condition were imaged every 10 min for a minimum of 16 h. Time-lapse images for each field of view were analysed and the fate of each mitotic cell was determined. Cell death was characterised by blebbing and dense cellular irregularities. Cells that left the field of view during the course of the experiment were not included in the analysis.

### Site-directed mutagenesis

An AKT1 ORFEXPRESS-Shuttle Clone was obtained from GeneCopoeia (A0022). Site-directed mutagenesis was performed to remove the stop-codon from the entry vector as this would enable it to be contiguous with the expression of a V5 tag once it is sub cloned into a pLX302 expression plasmid. To do this, primer sequences were designed using QuikChange Primer Design (Agilent Technologies) and reactions were performed using the QuikChange II site-directed mutagenesis kit (Agilent Technologies) according to the manufacturer's guidelines. After completion of the PCR, 40 μl of the PCR reaction was digested with *Dpn1* (New England BioLabs) for 1 h at 37°C to remove any template DNA that had not been mutagenised. Site-directed mutagenesis was also performed to introduce specific nucleotide changes/point mutations in the AKT1 entry vector to generate AKT-T308D/S473D.

### Transformation

50 μl ONE Shot TOP10 or ONE SHOT Stbl3 Chemically Competent *E.coli* (Thermo Fisher Scientific) were thawed on ice for each transformation. 5 μl plasmid DNA was added to the competent cells and mixed by flicking the tube before storing on ice for 30 min. Cells were then placed at 42°C for 30 seconds to enable incorporation of DNA and then placed on ice for 2 min. 250 μl of Super Optimal broth with Catabolite repression (SOC) medium at room temperature was added to each vial and placed in a 37°C orbital shaker for 2 h. After incubation, 20 μl and 200 μl of each transformation was placed on a pre-warmed agar plate containing the appropriate antibiotic and spread using ColiRoller Plating Beads (Millipore). Plates were wrapped in Parafilm (Thermo Fisher), inverted and placed in a 37°C incubator overnight. Plates were checked the next day for bacterial colonies and placed at 4°C until required.

### DNA extraction from transformed colonies

Transformed colonies were picked at random from the agar plates and grown at 37°C overnight in LB broth supplemented with the appropriate antibiotic in an orbital shaker at 225 rpm. The following day, bacteria were pelleted at 4000 rpm for 10 min in a desktop microcentrifuge. Supernatants were discarded and DNA was extracted using the QIAprep Spin Miniprep Kit (Qiagen) following the manufacturer's instructions. DNA sequences were verified with Sanger sequencing.

### Gateway cloning

Lentiviral vectors were generated using Gateway Technology (Thermo Fisher Scientific) according to the manufacturer's instructions. The AKT1 ORFEXPRESS-Shuttle Clone (A0022, GeneCopoeia was sub-cloned into the pLX302 lentiviral destination vector (Addgene) using the Gateway LR Clonase II enzyme mix. In brief, 5 μl reactions containing 70ng plasmid DNA, 150ng pLX302, 1 μl LR Clonase II Enzyme Mix and Tris EDTA (TE) buffer were mixed by vortexing and incubated at 25°C for 1 h. Reactions were incubated with 1 μl Proteinase K solution at 37°C for 10 min to terminate the reaction. 1 μl of each LR reaction was transformed into ONE SHOT Stbl3 Chemically Competent *E.coli* (Thermo Fisher Scientific) as described previously and success was checked using CMV-Forward and WPRE-Reverse primers. pCDH-puro-Myr-HA-AKT1 was a gift from Jialiang Wang (Addgene plasmid #46969), which was created as previously described [63]. DNA was extracted from the Myr-AKT1 pCDH lentiviral expression plasmid by inoculating LB-antibiotic broth with the glycerol stock and following the protocol for DNA extraction. DNA was then transformed into One Shot Stbl3 cells as previously described ready for transfection of packaging cells.

### Lentiviral transfection and transduction

Packaging cells (HEK293T) were plated in vented T25 flasks for 24 h to obtain 70% confluency. For each flask, the following were combined; 3 μg pLX302 vector, 0.3 μg VSV-G and 3 μg pCMV-Gag-Pol (10:1:10 ratio) in a total volume of 225 μl Opti-MEM. HEK293T were then transfected using the protocol from FuGENE with a FuGENE:DNA ratio of 3:1. In brief, 18.9 μl FUGENE was added to the DNA-OptiMEM mix and mixed by pipetting before incubation at room temperature for 7.5 min. 100 μl of the DNA-FUGENE-OptiMEM complex was added to the cells in 2 ml fresh media and left for 48 h. After 48 h, viral supernatants were harvested into a 15 ml falcon tube and centrifuged at 1500 rpm (365 xg) for 5 min to remove any cells before being passed through a 0.45 μm Minisart NML Syringe Filter (Sartorius). 2 ml DMEM media containing 30% (v/v) fetal bovine serum (Thermo Fisher Scientific) and 1% (v/v) penicillin/streptomycin (Thermo Fisher Scientific) were added to the HEK293T cells for a further 24 h, after which this was centrifuged and filtered as described above. 15,000 SKOV3 cells were plated in T25 flasks to obtain 50% confluence the day before transduction. The next day, media was removed and the filtered viral supernatants were added to the SKOV3 cells. SKOV3 were incubated with the supernatants for 72 h, adding the second filtered viral supernatant after the first 24 h. After 72 h, the media of SKOV3 was changed to contain 50ng/L Puromycin (Sigma-Aldrich) to select the cells that contain the integrated virus. HEK293T and SKOV3 cells were collected by trypsinisation for lysis and examination of protein expression by western blot to assess the efficiency of transfection and transduction.

### ATP assay

The CellTiter-Glo Luminescent Cell Viability assay (Promega) was used to measure ATP levels according to the manufacturer's protocol. Following mitotic cell isolation, populations of mitotic and interphase cells were counted in the presence of trypan blue and diluted to 200,000 cells/ml. 100 μl of each sample was plated in an opaque-walled 96-well plate in triplicate. Media alone was added in triplicate to allow the exclusion of background luminescence. 100 μl of the reconstituted substrate was added to each well in the dark and the plate was covered with foil and placed on a plate shaker for 2 min prior to incubation at room temperature for 10 min. The plate was read on a luminescent plate reader at a sensitivity of 190. Triplicate values were averaged and the culture media background was subtracted from the average of each experimental sample.

### Glutathione assay

Glutathione levels were measured using GSH-Glo Glutathione Assay (Promega) according to the manufacturer's instructions. Cells were diluted to 100,000 cells/ml. 50 μl cell suspension was placed in an opaque-walled 96-well plate in triplicate. 50 μl of freshly prepared GSH-Glo Reagent 2X Mix (10 μl luciferin-NT substrate, 10 μl glutathione-s-transferase with 480 μl GSH-Glo reagent) was added to each well in the dark and placed on a plate shaker for 30 seconds prior to incubation at room temperature for 30 min in the dark. After this, 100 μl/well of reconstituted Luciferin Detection Reagent was added and placed on a plate shaker for 30 seconds prior to incubation for 15 min and reading the luminescence. Triplicate values were averaged and the culture media background was subtracted from the average of each experimental sample.

### Glucose 6-phosphate assay

A Glucose 6-phosphate Assay Kit was purchased from Abcam and performed following the manufacturer's protocol. Cells were homogenised with ice-cold PBS and 50 μl were added to a 96-well plate with 50 μl reaction mix (46 μl assay buffer, 2 μl enzyme mix, 2 μl substrate mix). To measure the background, 50 μl of background mix (48 μl assay buffer, 2 μl substrate mix) was added to 50 μl PBS. Cells grown in glucose-free DMEM (Thermo Fisher Scientific) for 24 h were used as a negative control. The plate was incubated at room temperature for 30 min in the dark before measuring the OD at 450nm.

Further details regarding the methods are available as supplemental information.

## SUPPLEMENTARY MATERIALS FIGURES AND TABLES




