# Supplementary material for: Loss of PFKFB4 induces cell death in mitotically arrested ovarian cancer cells
Source: Oncotarget. 2017 Jan 31;8(11):17960–80. doi: 10.18632/oncotarget.14910 (PMC5392300; doi:10.18632/oncotarget.14910)
Supplement: Supplementary file 1 [file oncotarget-08-17960-s001.pdf]

Loss of PFKFB4 induces cell death in mitotically arrested ovarian cancer cells

SUPPLEMENTARY FIGURES AND TABLES

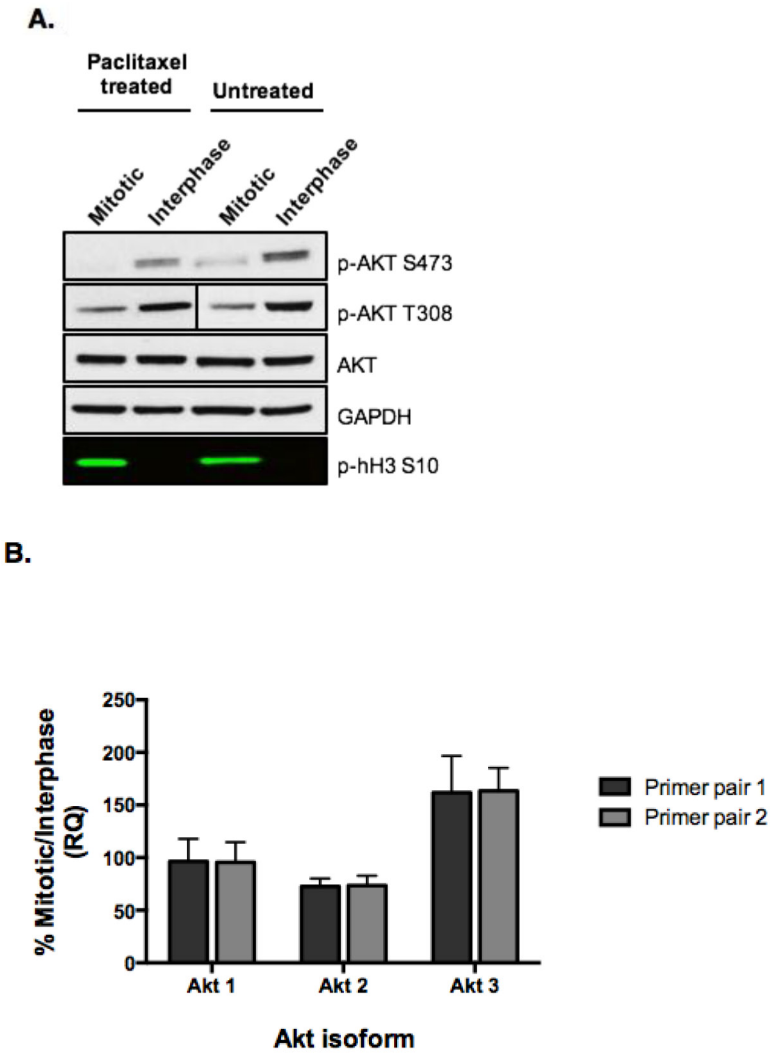

Supplementary Figure 1: related to Figure 1. A. SKOV3 cells were treated with 50 nM paclitaxel or DMSO vehicle control for 16 h prior to mitotic shake-off to isolate the mitotic population from interphase cells. AKT expression and phosphorylation were assessed by western blotting. GAPDH was used as a loading control and phosphor-histone H3 S10 as a mitotic marker. Displaying one of six experiments. **B.** After 16 h paclitaxel treatment, mitotically arrested and interphase cells were separated and total RNA was extracted from each population. Total RNA was reverse transcribed to cDNA, which was used to detect the expression of the AKT isoforms 1, 2 and 3 using two primer sets for each in a quantitative PCR.

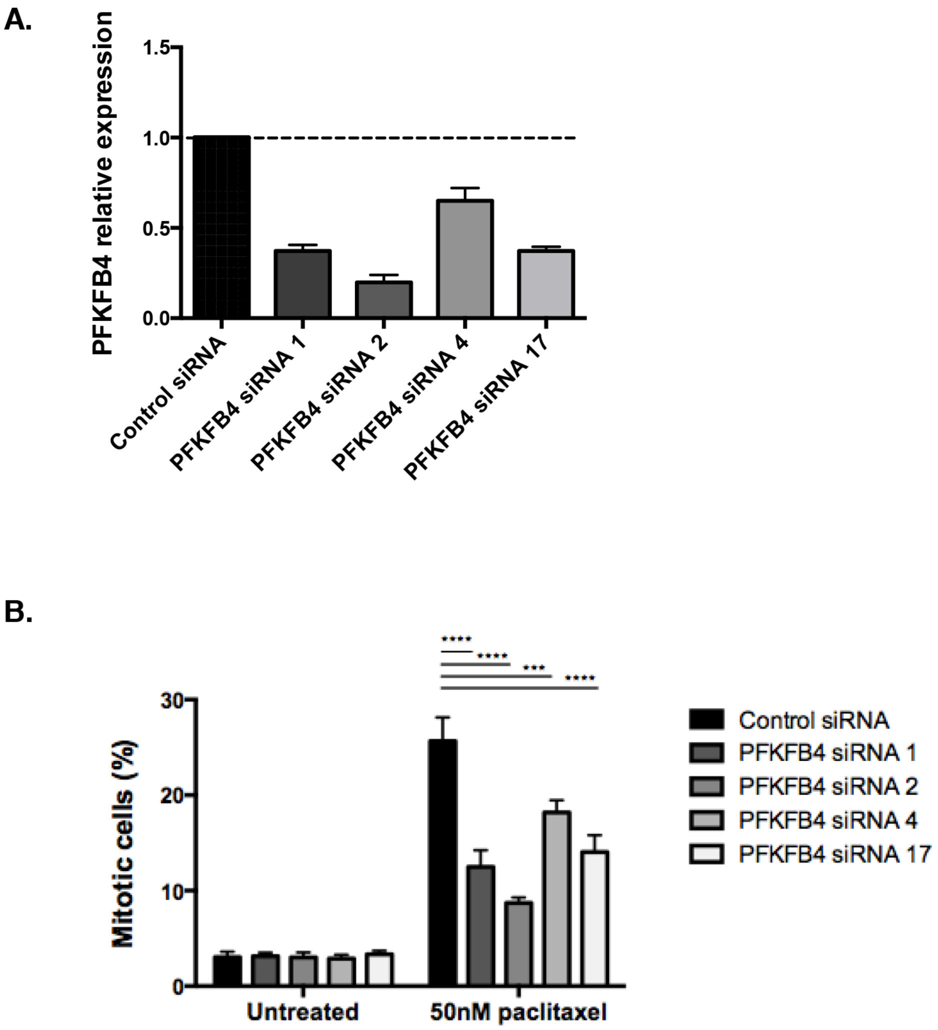

Supplementary Figure 2: related to Figure 4. A. siRNA-mediated depletion of *PFKFB4* with four different siRNA duplexes for 72 h in SKOV3 cells and quantification of *PFKFB4* mRNA expression using qPCR. **B.** SKOV3 cells transfected with four different siRNA duplexes targeting *PFKFB4* prior to 50 nM paclitaxel for 16 h and immunofluorescence. Cells were stained with DAPI and phospho-histone H3 S10 to quantify the percentage of mitotic cells.

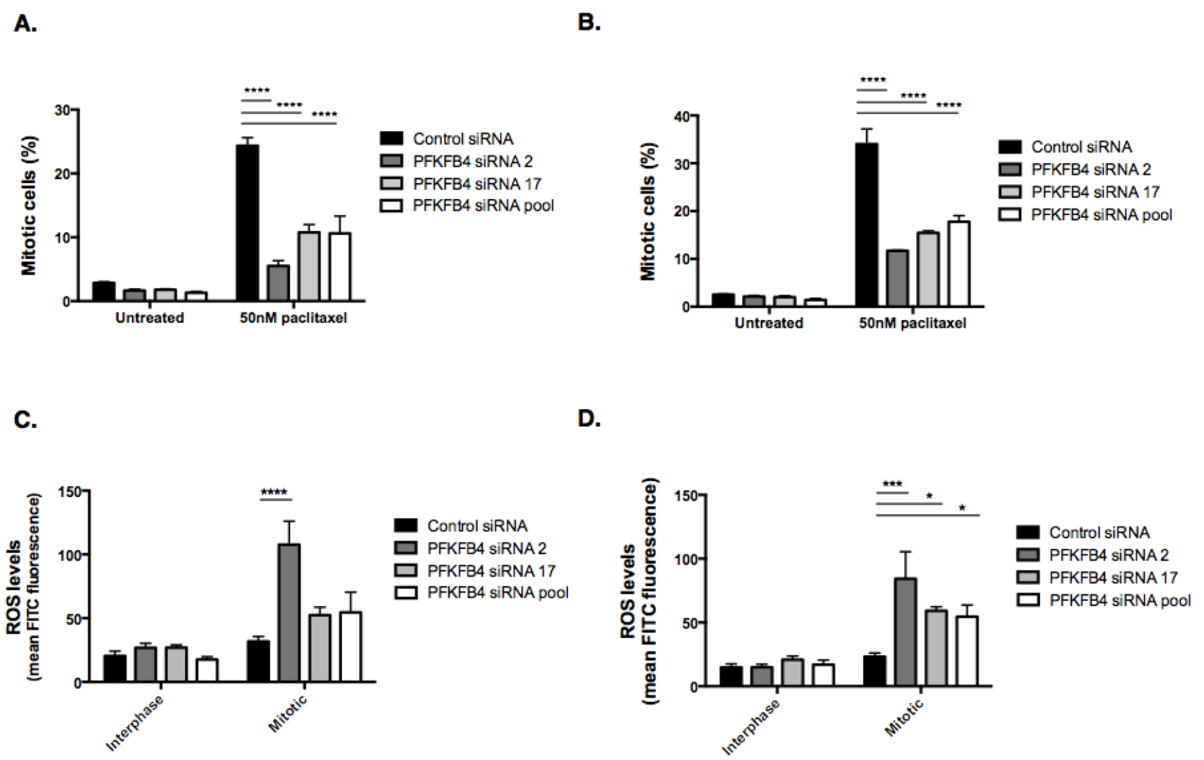

Supplementary Figure 3: related to Figure 10. SKOV3 cells were transfected with three different siRNA duplexes targeting PFKFB4 prior to 16 h 50 nM paclitaxel and mitotic shake-off. The percentage of mitotically arrested cells was quantified by phospho-histone H3 staining and flow cytometry and levels of ROS were measured by CM-H_2DCFDA staning and flow cytometry in T308D/S473D cells **A, C.** and Myr-AKT cells **B, D.**

Supplementary Table 1: Target genes that resulted in a PUMR/PMR ratio of 2 or more.

See Supplementary File 1

Supplementary Table 2: Primary antibodies for western blotting

Antibody against	Supplier	Dilution
GAPDH	Millipore	1:2500
pan-AKT	Abcam	1:1000
AKT1/2/3	Cell Signaling Technology	1:1000
phospho-AKT1 S473 (pan)	Cell Signaling Technology	1:1000
phospho-AKT1 T308 (pan)	Cell Signaling Technology	1:1000
phospho-histone H3 S10	Cell Signaling Technology	1:1000
phospho-p70S6K T389	Cell Signaling Technology	1:1000
phospho-GSK3β S9	Cell Signaling Technology	1:1000
phospho-PRAS40 T246	Cell Signaling Technology	1:1000
mTOR	Cell Signaling Technology	1:1000
RICTOR	Cell Signaling Technology	1:1000
AMPK	Cell Signaling Technology	1:1000
phospho –AMPK T172	Cell Signaling Technology	1:1000
ACC	Cell Signaling Technology	1:1000
phospho –ACC S79	Cell Signaling Technology	1:1000
AS160	Cell Signaling Technology	1:1000
phospho -AS160 T642	Cell Signaling Technology	1:1000
PTEN	Cell Signaling Technology	1:1000
PFKFB4	Abcam	1:250
PFKFB3	Proteintech	1:1000

Supplementary Table 3: Primer sequences for qPCR

Gene	Primers sequences for quantitative PCR		PrimerBank ID (if applicable)
	Forward Sequence	Reverse Sequence	
AKT1	5'- AGCGACGTGGCTATTGTGAAG -3'	5'- GCCATCATTCTTGAGGAGGAAGT -3'	62241012c1
AKT1	5'- GCTGAGATTGTGTCAGCCCT -3'	5'- TAATGTGCCCGTCCTTGTCC -3'	
AKT2	5'- ACCACAGTCATCGAGAGGACC -3'	5'- GGAGCCACACTTGTAGTCCA -3'	339895854c1
AKT2	5'- AGCATTTCATCCTGTGGGGG -3'	5'- CCAGAGGTGTTGCTACAGGG -3'	
AKT3	5'- TGTGGATTTACCTTATCCCCTCA -3'	5'- GTTTGGCTTTGGTCGTTCTGT -3'	332078467c1
AKT3	5'- TTTCTCCAAGTTGGGGGCTC -3'	5'- CCCCTCTTCTGAACCCAACC -3'	
PFKFB3	5‘-AGCCCGGATTACAAAGACTGC -3'	5‘-GGTAGCTGGCTTCATAGCAAC -3'	
PFKFB3	5'-GGCCGCATCGGGGGCGACTC-3'	5'-TTGCGTCTCAGCTCAGGGAC-3'	
PFKFB4	5'- GCCCAGTTCATCATCAGTGACCA -3'	5'- TCGTTGAGGACCTTCCACTG -3'	
PFKFB4	5‘-ACAGTGCATGTACCTCTGGATG -3'	5‘-TAATGCATCCAGTCCAGCAACT -3'	
ACTB	5'-CCTGGCACCCAGCACAAT-3'	5'-GCCGATCCACACGGAGTACT-3'	
GAPDH	5'- GCAAATTCCATGGCACCG-3'	5'- TCGCCCCACTTGATTTTGG -3'	

Supplementary Table 4: siRNA duplexes for transfections

Target mRNA	siRNA sequence	Catalog number	Purchased from
PFKFB4 1	GAGCGACCAUCUUUAAUUU	MU-006764-01-0002 D-006764-1, siGENOME	Dharmacon, GE Healthcare
PFKFB4 2	GAAAUGACCUACGAGGAAA	MU-006764-01-0002 D-006764-2, siGENOME	Dharmacon, GE Healthcare
PFKFB4 4	CAUCGUAUAUUACCUCAUG	MU-006764-01-0002 D-006764-4, siGENOME	Dharmacon, GE Healthcare
PFKFB4 17	GGGACAGGCCUCAGAACGU	MU-006764-01-0002 D-006764-17, siGENOME	Dharmacon, GE Healthcare
PFKFB3	GCCGCAUCGACUACCUGAUGAA	HSS107860 1299001, Stealth	Thermo Fisher Scientific
