# Supplementary material for: Loss of PFKFB4 induces cell death in mitotically arrested ovarian cancer cells
Source: Oncotarget. 2017 Jan 31;8(11):17960–80. doi: 10.18632/oncotarget.14910 (PMC5392300; doi:10.18632/oncotarget.14910)
Supplement: Supplementary file 2 [file oncotarget-08-17960-s002.docx]

**Supplementary Table 1. Target genes that resulted in a PUMR/PMR ratio of 2 or more**

| **Gene** | **PUDR** | **PDR** | **PUMR** | **PMR** | **PUMR/PMR** |
| --- | --- | --- | --- | --- | --- |
| CCR9 | 122.25 | 132.19 | 98.01 | 6.42 | 15.28 |
| WIF1 | 112.06 | 128.65 | 303.74 | 24.25 | 12.53 |
| TEX14 | 116.07 | 101.37 | 198.67 | 18.78 | 10.58 |
| CCR2 | 104.39 | 127.51 | 95.67 | 10.23 | 9.35 |
| XYLB | 101.82 | 118.52 | 99.83 | 12.32 | 8.10 |
| PLK1 | 100.93 | 98.80 | 139.26 | 17.88 | 7.79 |
| PACE-1 | 106.62 | 104.54 | 82.07 | 10.65 | 7.70 |
| MVD | 103.67 | 122.41 | 66.66 | 9.51 | 7.01 |
| GNB4 | 99.85 | 100.42 | 57.56 | 8.32 | 6.92 |
| MYO3A | 103.58 | 117.68 | 175.94 | 26.41 | 6.66 |
| WEE1 | 101.55 | 111.17 | 81.64 | 12.29 | 6.64 |
| ABI1 | 121.47 | 136.48 | 102.50 | 15.90 | 6.45 |
| PRPS1 | 107.19 | 109.87 | 211.93 | 33.44 | 6.34 |
| BLR1 | 120.99 | 107.61 | 179.35 | 28.65 | 6.26 |
| MAPK4 | 122.29 | 128.53 | 154.22 | 25.28 | 6.10 |
| PRKG2 | 104.99 | 120.26 | 61.77 | 10.32 | 5.99 |
| BCKDK | 102.08 | 105.76 | 236.15 | 41.63 | 5.67 |
| SQSTM1 | 114.74 | 117.06 | 189.39 | 34.08 | 5.56 |
| PITPNM3 | 105.80 | 117.89 | 135.96 | 24.72 | 5.50 |
| MPP2 | 106.68 | 98.96 | 56.90 | 11.27 | 5.05 |
| RPS6KA1 | 98.80 | 110.62 | 83.62 | 17.27 | 4.84 |
| MGC4796 | 112.63 | 117.21 | 266.88 | 55.84 | 4.78 |
| MIDORI | 107.17 | 126.74 | 287.25 | 60.51 | 4.75 |
| FKSG79 | 110.82 | 118.72 | 370.24 | 78.70 | 4.70 |
| CXCR3 | 107.99 | 131.34 | 361.37 | 77.70 | 4.65 |
| STK22C | 118.62 | 130.54 | 110.89 | 24.01 | 4.62 |
| MAP3K7 | 118.63 | 127.22 | 149.88 | 33.92 | 4.42 |
| GNAI1 | 102.45 | 111.29 | 20.15 | 4.57 | 4.41 |
| CAMK2B | 106.31 | 136.85 | 351.89 | 88.68 | 3.97 |
| AVPR2 | 102.54 | 100.56 | 88.71 | 22.84 | 3.88 |
| EPHB6 | 102.38 | 108.13 | 77.27 | 19.97 | 3.87 |
| CDKN3 | 102.08 | 100.82 | 18.90 | 4.97 | 3.80 |
| PFKFB4 | 97.02 | 133.00 | 76.84 | 20.75 | 3.70 |
| MATK | 95.02 | 135.86 | 93.77 | 25.37 | 3.70 |
| CSF1R | 100.57 | 113.92 | 294.74 | 82.19 | 3.59 |
| DEFB4 | 127.07 | 130.49 | 34.56 | 9.65 | 3.58 |
| PMVK | 108.82 | 124.58 | 192.31 | 54.31 | 3.54 |
| RIOK1 | 100.00 | 114.44 | 91.06 | 26.02 | 3.50 |
| TLR6 | 111.25 | 110.44 | 91.06 | 26.73 | 3.41 |
| EPHA7 | 110.44 | 109.50 | 122.28 | 35.97 | 3.40 |
| TLR4 | 93.12 | 86.30 | 65.67 | 19.36 | 3.39 |
| IHPK3 | 108.85 | 108.97 | 153.21 | 45.28 | 3.38 |
| RPS6KA3 | 106.94 | 120.49 | 229.40 | 68.71 | 3.34 |
| CARD10 | 101.20 | 103.37 | 161.62 | 49.29 | 3.28 |
| SMG1 | 96.68 | 90.22 | 52.89 | 16.70 | 3.17 |
| CDC2L2 | 108.50 | 123.11 | 194.72 | 61.54 | 3.16 |
| DUSP4 | 102.36 | 121.50 | 116.20 | 36.90 | 3.15 |
| KSR2 | 112.83 | 125.68 | 102.68 | 33.56 | 3.06 |
| TJP2 | 105.32 | 109.69 | 428.13 | 140.83 | 3.04 |
| DGKI | 104.89 | 137.11 | 224.87 | 74.56 | 3.02 |
| DGKE | 100.42 | 97.56 | 121.18 | 40.26 | 3.01 |
| MAP3K10 | 110.56 | 136.70 | 397.32 | 132.95 | 2.99 |
| RAPGEF3 | 105.59 | 110.97 | 88.15 | 29.55 | 2.98 |
| MAP2K1 | 123.53 | 135.24 | 241.84 | 81.10 | 2.98 |
| KALRN | 104.08 | 103.79 | 176.07 | 59.06 | 2.98 |
| BUB1 | 88.81 | 104.79 | 126.62 | 42.49 | 2.98 |
| CAMKK2 | 103.65 | 95.88 | 155.40 | 52.30 | 2.97 |
| FLT4 | 100.71 | 102.26 | 117.74 | 39.92 | 2.95 |
| CSNK1G3 | 106.80 | 128.91 | 117.90 | 40.09 | 2.94 |
| PLK3 | 108.22 | 132.73 | 177.95 | 60.52 | 2.94 |
| C6ORF199 | 106.00 | 112.28 | 90.98 | 31.28 | 2.91 |
| RPS6KA4 | 106.09 | 120.51 | 97.10 | 33.40 | 2.91 |
| MAP3K13 | 103.60 | 109.29 | 114.96 | 39.63 | 2.90 |
| FKSG83 | 95.55 | 98.12 | 131.52 | 45.37 | 2.90 |
| DGKZ | 102.91 | 124.50 | 171.91 | 59.56 | 2.89 |
| PPP2CB | 105.92 | 119.32 | 109.28 | 37.95 | 2.88 |
| CINP | 108.50 | 123.45 | 54.00 | 18.93 | 2.85 |
| EPHA4 | 109.71 | 121.71 | 45.30 | 15.88 | 2.85 |
| PRKR | 104.08 | 98.50 | 189.45 | 66.56 | 2.85 |
| GRK4 | 103.49 | 107.14 | 37.12 | 13.09 | 2.83 |
| CNR2 | 108.11 | 108.52 | 261.08 | 92.42 | 2.83 |
| ADRB3 | 106.10 | 108.82 | 175.30 | 62.08 | 2.82 |
| PRPS1L1 | 106.98 | 121.47 | 54.78 | 19.57 | 2.80 |
| MAGI-3 | 104.25 | 115.99 | 298.04 | 107.71 | 2.77 |
| PRKCB1 | 100.73 | 102.88 | 84.01 | 30.42 | 2.76 |
| TRIB2 | 100.23 | 119.05 | 45.82 | 16.70 | 2.74 |
| MERTK | 102.88 | 111.61 | 305.54 | 112.62 | 2.71 |
| NPR1 | 110.03 | 108.00 | 105.01 | 38.74 | 2.71 |
| BTK | 105.22 | 111.74 | 58.68 | 21.69 | 2.71 |
| PXK | 102.79 | 122.34 | 193.71 | 72.19 | 2.68 |
| CXCL12 | 103.94 | 101.90 | 44.80 | 16.75 | 2.67 |
| MAP3K1 | 101.13 | 110.30 | 73.30 | 27.61 | 2.65 |
| AGTR2 | 108.96 | 104.61 | 91.72 | 34.75 | 2.64 |
| NEK7 | 105.63 | 124.36 | 192.22 | 73.09 | 2.63 |
| FGR | 103.99 | 105.69 | 26.69 | 10.16 | 2.63 |
| MAP3K8 | 106.79 | 116.12 | 89.93 | 34.44 | 2.61 |
| RET | 104.21 | 123.21 | 184.00 | 70.50 | 2.61 |
| CDADC1 | 97.32 | 92.25 | 35.69 | 13.74 | 2.60 |
| CX3CR1 | 109.20 | 103.25 | 57.88 | 22.37 | 2.59 |
| GALK1 | 106.90 | 120.94 | 82.87 | 32.08 | 2.58 |
| SMAD7 | 105.31 | 126.64 | 183.47 | 71.07 | 2.58 |
| CCR6 | 109.44 | 133.95 | 153.68 | 59.73 | 2.57 |
| SYK | 105.45 | 113.17 | 129.83 | 51.45 | 2.52 |
| FZD9 | 116.71 | 120.31 | 68.07 | 26.99 | 2.52 |
| ULK2 | 106.65 | 118.47 | 36.61 | 14.62 | 2.50 |
| MYLK | 104.77 | 128.37 | 80.18 | 32.19 | 2.49 |
| MAST3 | 103.49 | 140.97 | 87.63 | 35.74 | 2.45 |
| SPA17 | 106.64 | 119.48 | 30.30 | 12.38 | 2.45 |
| PHKG1 | 106.32 | 112.21 | 103.19 | 42.41 | 2.43 |
| GIT2 | 104.20 | 115.91 | 84.43 | 34.95 | 2.42 |
| GLP1R | 102.17 | 107.07 | 122.45 | 51.03 | 2.40 |
| MAP3K12 | 102.16 | 117.59 | 123.46 | 51.64 | 2.39 |
| CALCA | 104.91 | 120.61 | 384.71 | 161.69 | 2.38 |
| ACK1 | 97.31 | 100.93 | 246.06 | 103.79 | 2.37 |
| PTPRJ | 99.64 | 111.90 | 232.41 | 98.04 | 2.37 |
| ADRBK2 | 97.77 | 103.30 | 111.55 | 47.22 | 2.36 |
| DDR2 | 99.87 | 78.26 | 46.97 | 19.97 | 2.35 |
| PRKCN | 102.79 | 109.63 | 80.50 | 34.30 | 2.35 |
| MAP3K2 | 96.46 | 113.32 | 202.90 | 86.86 | 2.34 |
| FPRL2 | 104.10 | 108.10 | 89.29 | 38.40 | 2.33 |
| GSG2 | 101.49 | 123.35 | 152.01 | 65.61 | 2.32 |
| FY | 105.86 | 123.60 | 319.32 | 138.13 | 2.31 |
| PLK2 | 108.49 | 125.37 | 124.09 | 53.71 | 2.31 |
| DGKQ | 102.93 | 128.27 | 138.75 | 60.15 | 2.31 |
| ARAF1 | 106.37 | 119.50 | 11.84 | 5.15 | 2.30 |
| PTK2 | 107.01 | 95.40 | 40.38 | 17.59 | 2.30 |
| GNAI3 | 111.72 | 126.85 | 90.82 | 39.65 | 2.29 |
| SNF1LK | 107.60 | 112.32 | 94.45 | 41.25 | 2.29 |
| YES1 | 101.47 | 103.59 | 147.84 | 64.66 | 2.29 |
| PKN3 | 99.23 | 107.52 | 99.35 | 43.68 | 2.27 |
| CHRM3 | 108.51 | 113.20 | 78.05 | 34.39 | 2.27 |
| TLK1 | 105.76 | 116.67 | 138.99 | 61.32 | 2.27 |
| TAO1 | 98.46 | 115.65 | 125.29 | 55.74 | 2.25 |
| PDGFRA | 110.93 | 103.34 | 85.82 | 38.19 | 2.25 |
| CDC7 | 106.87 | 109.13 | 67.32 | 30.04 | 2.24 |
| STK22B | 113.49 | 128.33 | 250.50 | 112.38 | 2.23 |
| TSKS | 111.65 | 133.10 | 132.34 | 59.48 | 2.22 |
| TGFBR2 | 99.45 | 113.81 | 107.10 | 48.36 | 2.21 |
| CSNK1A1 | 97.58 | 103.69 | 137.05 | 62.05 | 2.21 |
| JAK1 | 95.27 | 113.65 | 52.77 | 23.91 | 2.21 |
| TRIM | 100.35 | 111.52 | 79.36 | 36.04 | 2.20 |
| STK6 | 111.27 | 109.92 | 169.81 | 77.48 | 2.19 |
| PRPF4B | 97.57 | 103.00 | 41.55 | 18.98 | 2.19 |
| MGC26597 | 99.16 | 110.22 | 70.74 | 32.35 | 2.19 |
| GCK | 103.24 | 114.50 | 50.33 | 23.07 | 2.18 |
| MST4 | 101.75 | 116.48 | 131.01 | 60.28 | 2.17 |
| NME1 | 98.71 | 102.76 | 80.36 | 37.09 | 2.17 |
| LYN | 107.71 | 118.59 | 140.02 | 65.07 | 2.15 |
| COL4A3BP | 109.65 | 115.31 | 360.32 | 167.53 | 2.15 |
| PTK9 | 103.62 | 105.01 | 109.14 | 50.82 | 2.15 |
| ASK | 103.90 | 106.23 | 57.92 | 26.98 | 2.15 |
| BAI2 | 95.57 | 106.92 | 76.78 | 35.83 | 2.14 |
| DRD1 | 108.62 | 127.18 | 172.27 | 80.71 | 2.13 |
| NEK11 | 101.71 | 126.62 | 81.08 | 38.25 | 2.12 |
| JAK2 | 110.90 | 126.93 | 166.52 | 78.91 | 2.11 |
| DYRK3 | 110.62 | 116.17 | 130.67 | 61.98 | 2.11 |
| MAPKAPK3 | 98.66 | 93.06 | 127.48 | 60.77 | 2.10 |
| GRK1 | 107.58 | 131.28 | 333.77 | 159.15 | 2.10 |
| GUCY2D | 107.24 | 114.74 | 88.22 | 42.21 | 2.09 |
| MARK2 | 107.18 | 130.15 | 117.71 | 56.33 | 2.09 |
| SRC | 98.48 | 103.45 | 101.41 | 48.66 | 2.08 |
| RIPK3 | 93.94 | 102.06 | 158.27 | 76.21 | 2.08 |
| LATS2 | 108.00 | 127.72 | 105.60 | 50.88 | 2.08 |
| CNKSR1 | 101.05 | 122.34 | 148.82 | 71.71 | 2.08 |
| CASK | 106.40 | 111.90 | 76.33 | 36.85 | 2.07 |
| MYO3B | 99.88 | 106.30 | 110.46 | 53.38 | 2.07 |
| TLR3 | 103.20 | 100.36 | 85.52 | 41.35 | 2.07 |
| LYK5 | 106.30 | 116.91 | 175.63 | 85.20 | 2.06 |
| ITK | 121.59 | 127.92 | 34.51 | 16.83 | 2.05 |
| STK22D | 98.19 | 122.69 | 122.63 | 59.85 | 2.05 |
| STK38L | 106.87 | 108.72 | 129.13 | 63.04 | 2.05 |
| PFKL | 119.25 | 126.91 | 110.10 | 53.76 | 2.05 |
| VRK1 | 101.86 | 116.87 | 138.47 | 68.29 | 2.03 |
| PKIB | 100.36 | 106.17 | 146.98 | 73.27 | 2.01 |
| RPS6KB1 | 105.84 | 117.54 | 273.36 | 136.39 | 2.00 |
| FASTK | 104.93 | 110.92 | 122.67 | 61.31 | 2.00 |

**PUMR** The mitotic index following gene depletion in untreated cells divided by the mitotic index following non-targeting siRNA control treatment in untreated cells

**PMR** The mitotic index following gene depletion in paclitaxel treated cells divided by the mitotic index following non-targeting siRNA control treatment in paclitaxel treated cells

**PUDR** The DNA content following gene depletion in untreated cells divided by the mitotic index following non-targeting siRNA control treatment in untreated cells

**PDR** The DNA content following gene depletion in paclitaxel treated cells divided by the mitotic index following non-targeting siRNA control treatment in paclitaxel treated cells

DNA content was measured using Hoechst staining

Mitotic index was quantified using anti-phospho-histone H3 S10
